# Current Overview of Corrosion Inhibition of API Steel in Different Environments

**DOI:** 10.1021/acsomega.4c01999

**Published:** 2024-06-15

**Authors:** Víctor Díaz-Jiménez, Giselle Gómez-Sánchez, Natalya Victorovna Likhanova, Paulina Arellanes-Lozada, Octavio Olivares-Xometl, Irina V. Lijanova, Janette Arriola-Morales

**Affiliations:** † Facultad de Ingeniería Química, Benemérita Universidad Autónoma de Puebla, Av. San Claudio y 18 Sur, Ciudad Universitaria, Col. Jardines de San Manuel, Puebla 72570, México; ‡ Dirección de Investigación, Instituto Mexicano del Petróleo, Eje Central Lázaro Cárdenas No. 152, Col. San Bartolo Atepehuacan, Ciudad de México 07730, México; § CIITEC, Instituto Politécnico Nacional, Cerrada Cecati S/N, Colonia Santa Catarina de Azcapotzalco, Ciudad de México 02250, Mexico

## Abstract

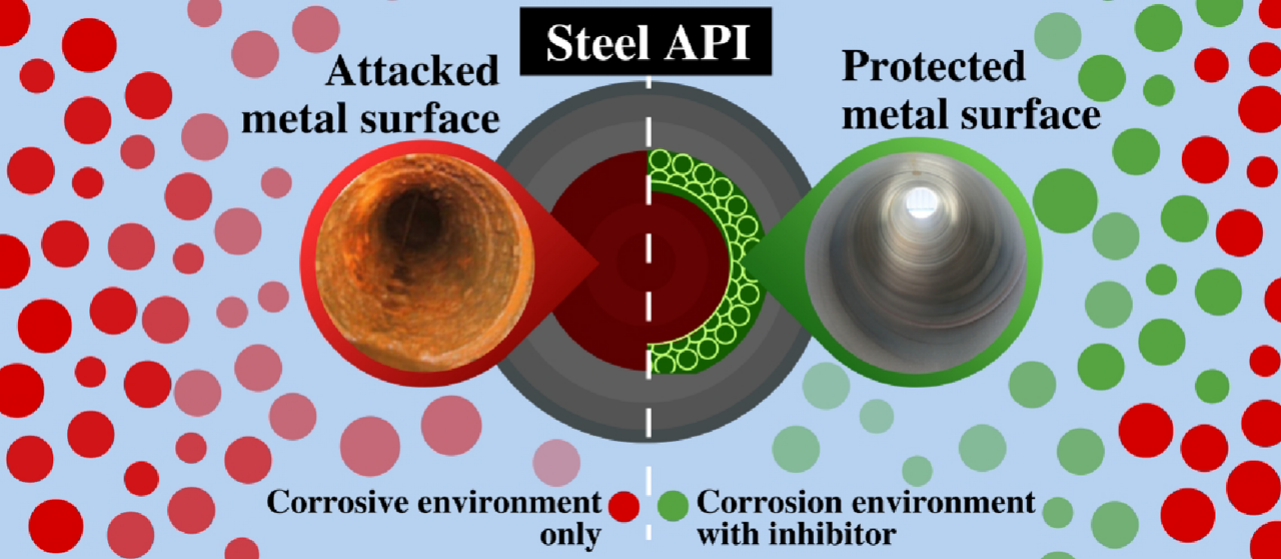

API (American Petroleum Institute) steels are the most employed metal alloys in the oil industry due to their outstanding mechanical properties; however, their protection is considered as an imperative matter because of their corrosion damage vulnerability when exposed to different surroundings that provoke a rate increase in the concomitant redox reactions. This problematic situation becomes more relevant when the generation and/or use of one or various aqueous corrosive environments occur, in addition to process conditions, the result of which is extremely difficult to be controlled. For these reasons, the internal and external protection of exposed metallic systems are considered as a fundamental concern, where internal corrosion is often controlled through the addition of corrosion inhibitors (CIs). The present review analyzes researchers’ contributions in the last years to the study and evaluation of CIs for API steel in different corrosive media featuring HCl, H_2_SO_4_, H_3_NSO_3_H, CO_2_, H_2_S, NaCl, and production water under different temperature and flow conditions. Different CIs derived from plant extracts, drugs, nanoparticles, or ionic liquids, mainly destined for acid media, were found. Throughout the review, an exhaustive analysis of inhibition process results is carried out based on gravimetric and/or electrochemical techniques that consider the weight loss of the metallic material and electrical behavior (current density, resistance, capacitance, frequency, impedance, etc.). Likewise, the results of computational analyses and those of surface analysis techniques were taken into account to reinforce the study of CIs.

## 1 Introduction

As energy needs grow in different industrial sectors, the use of fossil fuels continues to be the main supply energy source. This energy demand has promoted the extraction of crude oil, which is heavier and heavier. As a consequence, the recovery, transport, and processing means are affected by the formation of more aggressive corrosive media like systems that have the presence of hydrosulfuric acid, hydrochloric acid, and carbon dioxide, among others. In combination with variables such as temperature, pressure, and flow rate, these corrosive media become more complex, thus affecting the steel lifespan.^1,2^ Despite the advance in the development of materials with high mechanical resistance such as polymers, composites, ceramic materials, epoxy resins, etc., they are not suitable for industrial purposes because of the lack of mechanical resistance properties and low costs that offer metal alloys that are commonly employed in the oil industry. It is known, that the alloys that are industrially used are produced under international specifications according to operation design, with variations in their chemical composition and microstructure. For decades, the oil industry has employed high-strength, low-alloy steel (HSLAs), where API (American Petroleum Institute) steels belong to an HSLA subcategory. In this industry, the use of API steel is common for the manufacturing of pipelines, storage tanks, heat exchangers, and distillation columns, among others. This is because this material possesses excellent properties such as tractive resistance, tenacity, high impact energy, good weldability, resistance to uniform and localized corrosion, etc. Due to the foregoing, this type of steel is common in different industries (food, metallurgical, chemical, petrochemical, etc.).^1,2^ Specifically, API 5L steel is employed in the manufacturing of welded and seamless pipes for the transport of gas, water, and oil,^3^ where its classification X can vary according to the grade as X42, X46, X52, etc. and whose chemical composition is characterized by low carbon content as shown in Table 1.^4−6^

**Table 1:** Required Chemical Composition and Minimum Values of Mechanical Properties of Different Grades of API 5L Steel

	Typical composition %				Minimum yield strength		Minimum tensile strength	
API steel grade	C	Mn	P	S	MPa	kPsi	MPa	kPsi
A	0.22	0.90	0.03	0.03	207	30	331	48
B	0.26	1.20	0.03	0.03	241	35	414	60
X42	0.26	1.30	0.03	0.03	290	42	414	60
X46	0.26	1.40	0.03	0.03	317	46	434	63
X52	0.26	1.40	0.03	0.03	359	52	455	66
X56	0.26	1.40	0.03	0.03	386	56	490	71
X60	0.26	1.40	0.03	0.03	414	60	517	75
X65	0.26	1.45	0.03	0.03	448	65	531	77
X70	0.26	1.65	0.03	0.03	483	70	565	82

Even when the physicochemical properties of API 5L steel are ideal for its use at industrial level, these are affected when the material is exposed to aqueous^7^ or nonaqueous^8,9^ corrosive media that inflict irreversible damage caused by “corrosion”. Due to the fact that for the industry, the integrity of API 5L steel is paramount, integral studies have been put into action (cathodic protection, epoxy coating, stronger alloys, CIs, etc.) to protect it and extend its lifespan.^10,11^ As for the oil industry, both the right selection of the steel alloy and its protection method are crucial, where the CIs play a major role.^10,11^

## 2 Costs Generated by Corrosion

The oil and natural gas industry is a reference of the economic and technological development in many countries and represents almost 8% of their economy.^12^ This industry consists of different sectors such as exploration, transport, production, and refining, among others. Since this is a very influential economic activity, it is necessary that all the factors that affect the production process be thoroughly understood and solved because the corrosion damage caused by aggressive media is a tangible problem that has seen attempts to be trimmed, worked out, and controlled through effective management and monitoring.

**Figure 1:**
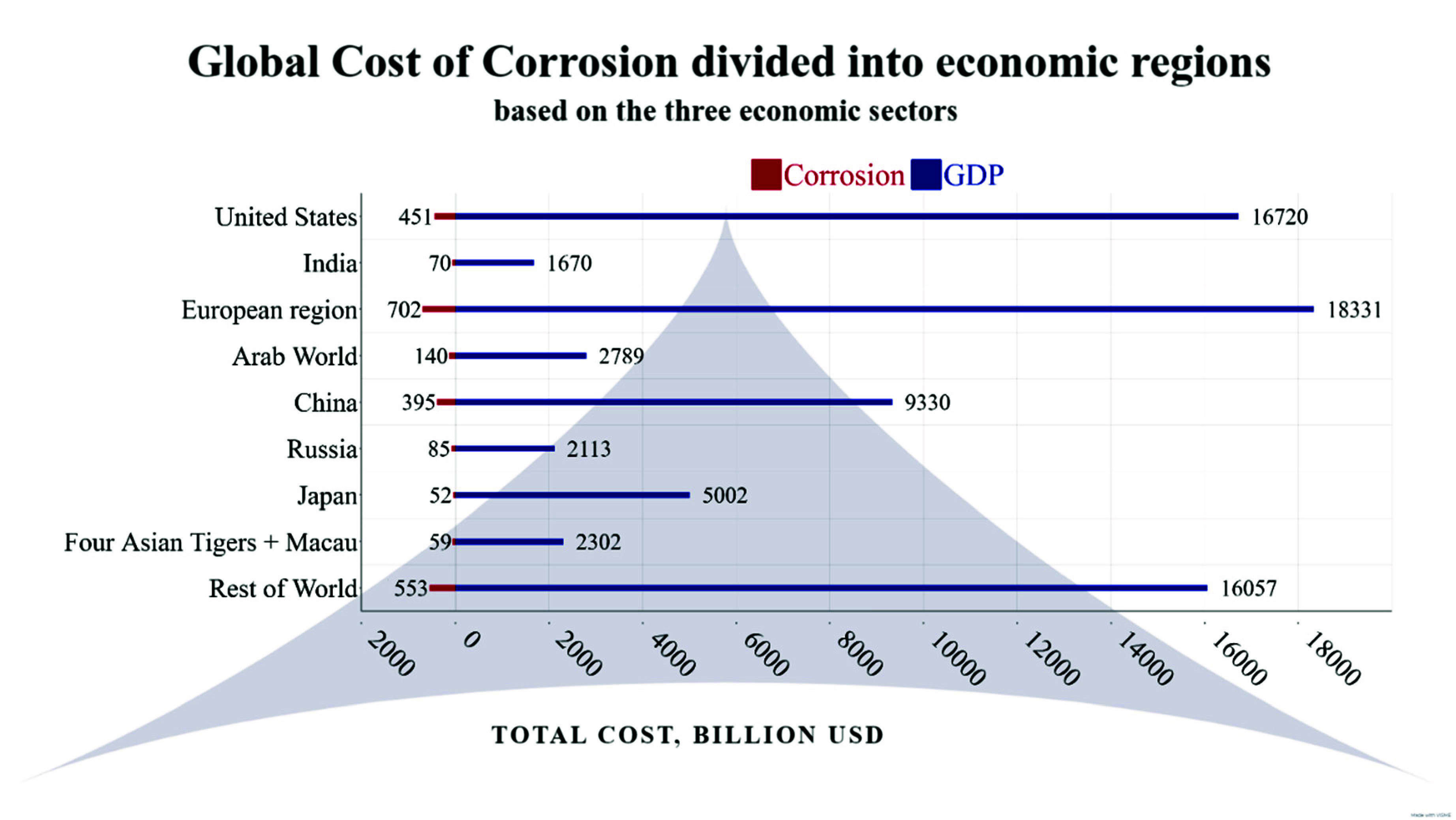
Comparative of total costs per GDP and corrosion in different parts of the world.

At an industrial level, corrosion damage of metal alloys includes direct and indirect costs that exert an important impact on both production processes and human life.^7^ In the literature, it has been reported that neglecting this matter can generate approximate total costs of 2.5 billion dollars worldwide that are equivalent to 3.4% of the global gross domestic product (GDP) as shown in Figure 1.^13^

Likewise, corrosion damage implies additional costs stemming from indirect expenses that include individual safety and environmental consequences, which in turn consider other indirect costs such as maintenance, storing, transport expenses, pollution/product loss, repackaging, production stops, plant shutdown, winding down of the process efficiency, and plant equipment overdesign, which requires more expensive and overqualified materials.^14^

## 3 Corrosion Inhibitors

It is known that corrosion of steel alloys is a natural phenomenon, spontaneous and irreversible, whose mitigation requires, mainly, the control of the oxidation (Reaction 1) and reduction (Reaction 2) reactions that take place on the metallic surface; due to the fact that the electrons generated in Reaction 1 are used in Reaction 2 (electroneutrality), both reactions occur simultaneously and with the same rate. These reactions can be distributed all over the steel surface (microcells), producing uniform corrosion or localized corrosion when on the metal there are specific cathodic and anodic sites:^15^
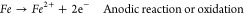
1
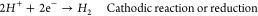
2

**Figure 2:**
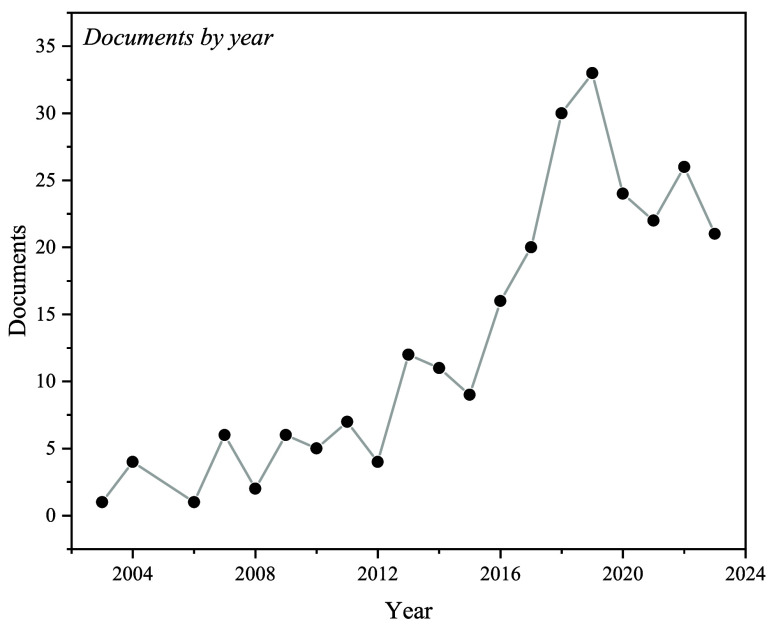
Research trend about API steel corrosion inhibitors in the last 20 years worldwide.

From the available methods for mitigating corrosion in industry, the use of CIs is a viable option for controlling the internal corrosion of systems that transport oil and its derivatives, where the formation of corrosive media is unavoidable. Figure 2 shows the interest evolution in the last 20 years in the topic of CIs as a method for reducing the corrosion of API steels, which confirms the relevance of the study of CIs for a steel type that is widely employed in the industry. The advantages of developing and proposing new CIs for API steels are their easy application, low cost, and high efficiency, being this field where the use of CIs has been diversified.^16^

The addition of CIs retards anodic or cathodic reactions that occur as a consequence of the attack of medium corrosive species through the adsorption of inhibitors on a metallic substrate. The corrosion inhibition of API steels can be achieved by means of inorganic inhibitors such as hydrazine, nitrates, pigments, or metal nanoparticles, which usually are excellent options within wide temperature intervals and extended times. However, the use of organic CIs has prevailed, despite being a more expensive option, because these chemical compounds are less toxic than inorganic CIs.^17^

The family of inorganic CIs is a big one, where compounds with functional groups like amines or amides, imidazolines, azoles, benzopyranones, ionic liquids (ILs) and their derivatives, among others have been employed. Their mechanism is based, generally, on competition against water molecules and aggressive ions for available sites on the steel surface. This phenomenon takes place through adsorption mechanisms by electrostatic interactions (physisorption) or by sharing electrons and forming chemical bonds between the CI molecules and the metal (chemisorption) through heteroatoms such as O, N, P, or S.^18^

During the last years, environmental and health risks associated with the use of synthetic inorganic and organic inhibitors have prompted the growing necessity of developing “green” CIs (e.g., based on plant extracts, biopolymers, or carbohydrates) capable of offering maximal metal protection with minimal impact on human beings and nature. The requisites for a chemical product to be approved as green CI have been established by legislative bodies like the Paris Commission (PARCOM) and the Registration, Evaluation, Authorization, and Restriction of Chemicals (REACH), which have established that green CIs must be nonbioaccumulative, biodegradable, and with zero or very low marine toxicity level.^19^

In general, the common goal in most research studies on CIs is that these compounds have to be highly efficient in the applied aqueous medium, thus retarding the steel corrosion rate. For this reason, all the studies on CIs consider the nature of substituent groups, electron cloud, presence of one or more functional groups, structural characteristics, and medium type.^20^ As a consequence, it has been reported that the inhibition efficiency (*IE*) of CIs has a close relationship with their concentration, corrosive medium type, pH, temperature and flow rate, among other factors.^21^

Even when the chemical structure of a CI is unique and totally different from others, the evaluation of their *IE* includes weight loss (WL) and electrochemical [linear polarization resistance (LPR), potentiodynamic polarization (PDP), electrochemical impedance spectroscopy (EIS), etc.] techniques, where international norms [American Society for Testing and Materials (ASTM) or Association for Materials Protection and Performance (AMPP)] play a major role in this matter. In addition to the corrosion evaluation methods, different surface analysis (SEM–EDS, FTIR, XPS, CA, ATR-IR, etc.) and/or computational (DFT and MD) techniques have been fundamental tools for confirming the protection properties of CIs.^22^

### 3.1 Corrosion Inhibition of Acidic Media

#### 3.1.1 Corrosion Inhibition of Hydrochloric Acid Environments

Hydrochloric acid (HCl) is a strong monoprotic acid that can be totally dissociated into H^+^ and Cl^–^. For this reason, the different types of commonly employed API steel undergo serious corrosion in HCl aqueous systems, forming ferrous chloride as the main corrosion product (Reaction 3). This chemical compound is not capable of forming a passivating film due to the high solubility in the low medium pH. This phenomenon has a relationship with the Cl^–^ ions, which are highly active and destroy the passive layer of the metal surface, provoking localized corrosion by “pitting” and “cracking” due to higher activity on the active sites of the steel surface.^6,23^ This phenomenon limits the selection of steels capable of resisting this type of medium, with respect to others. Due to the foregoing, metal alloys such as titanium, zirconium, tantalum, or nickel are frequently used because of their tendency for passivation and/or a strong thermodynamic stability:^24^
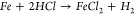
3

Despite the vulnerability of steel in HCl media, this acid is widely used at industrial level as cleaning, decalcifying, and pickling agent,^25,26^ as well as in acidizing processes of oil wells.^27^ In storing, transport, and refining of chemical products and/or crude oil, the operation occurs under a variety of corrosive conditions that deteriorate the metal surfaces of petrochemical process equipment;^26,28^ the foregoing provokes different and complex corrosion mechanisms that make unavoidable the use of CIs to retard the anodic dissolution of steel.^29^

In the past decade, natural-origin CIs for API steel intended to be exposed to HCl have been developed such as plant extracts,^25,30^ carbohydrates,^31,32^ and biopolymers,^26,33,34^ including organic/inorganic nanocomposites.^35^ Particularly, the proposals based on plant extracts have become popular due to the wide variety of organic active compounds that have outstanding features such as biodegradability, bioavailability, sustainability, and nontoxicity; the high availability of this raw matter implies an additional benefit that is reflected in the diminution of synthesis costs.^25,30,31^

Table 2 shows some CIs that were obtained from plant extracts and evaluated by employing different API steels. For example, Nikitasari et al.^25^ used the extract of a group of *Eucheuma* algae as CI of API 5L A steel in 1 M HCl. The authors attributed the inhibiting effect to the content of polyphenols, which can form complexes with the metal surface and reduce the charge transfer between the metal and the corrosive medium. Additionally, other studies have shown that algae belonging to this species present high antioxidant capacity due to their carotenoid content.^36^ In this context, it was observed that the *IE* was a function of the immersion time interval from 0 to 30 min, where the longer the time, the higher the extract adsorption, independent of the temperature.

**Table 2:**
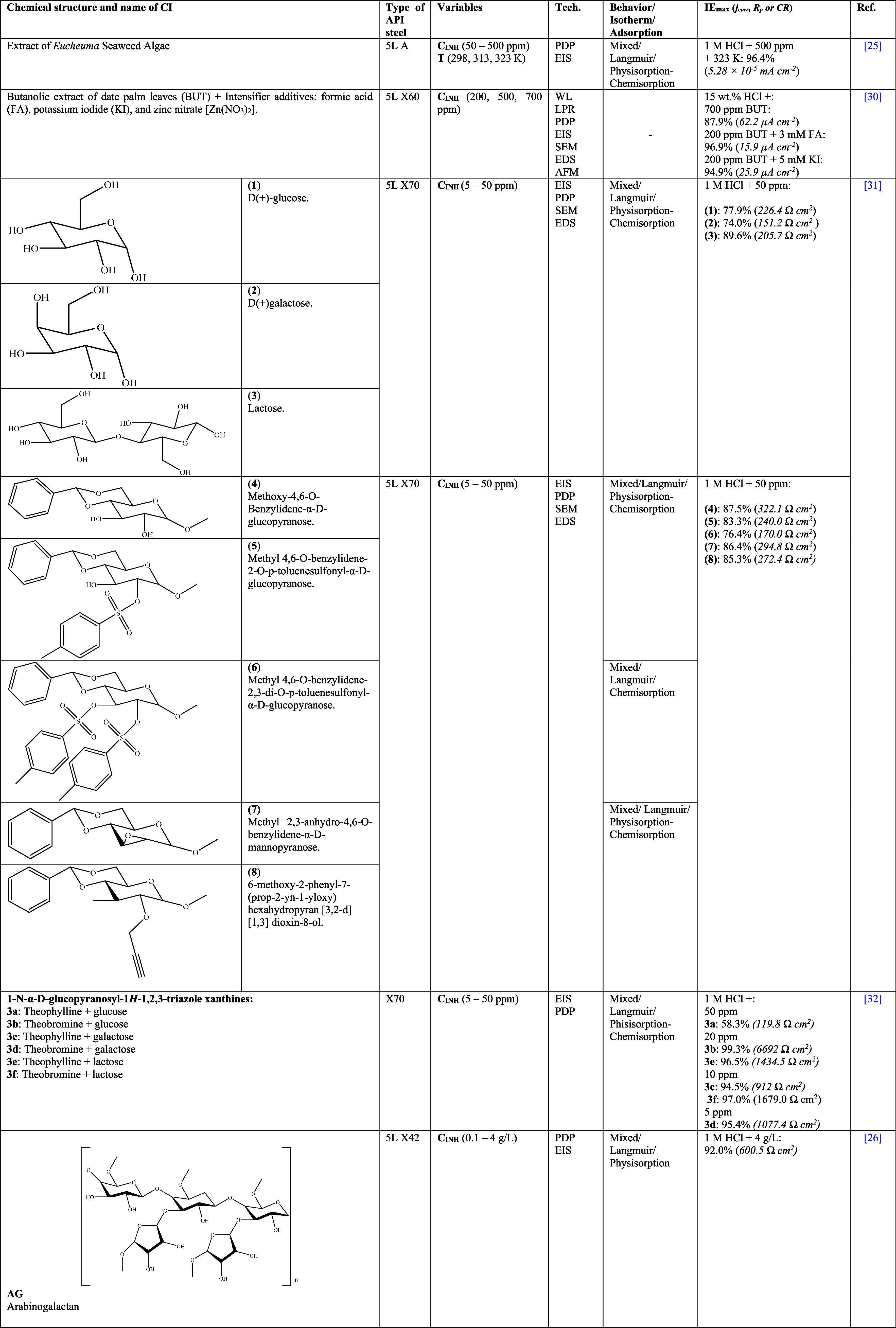

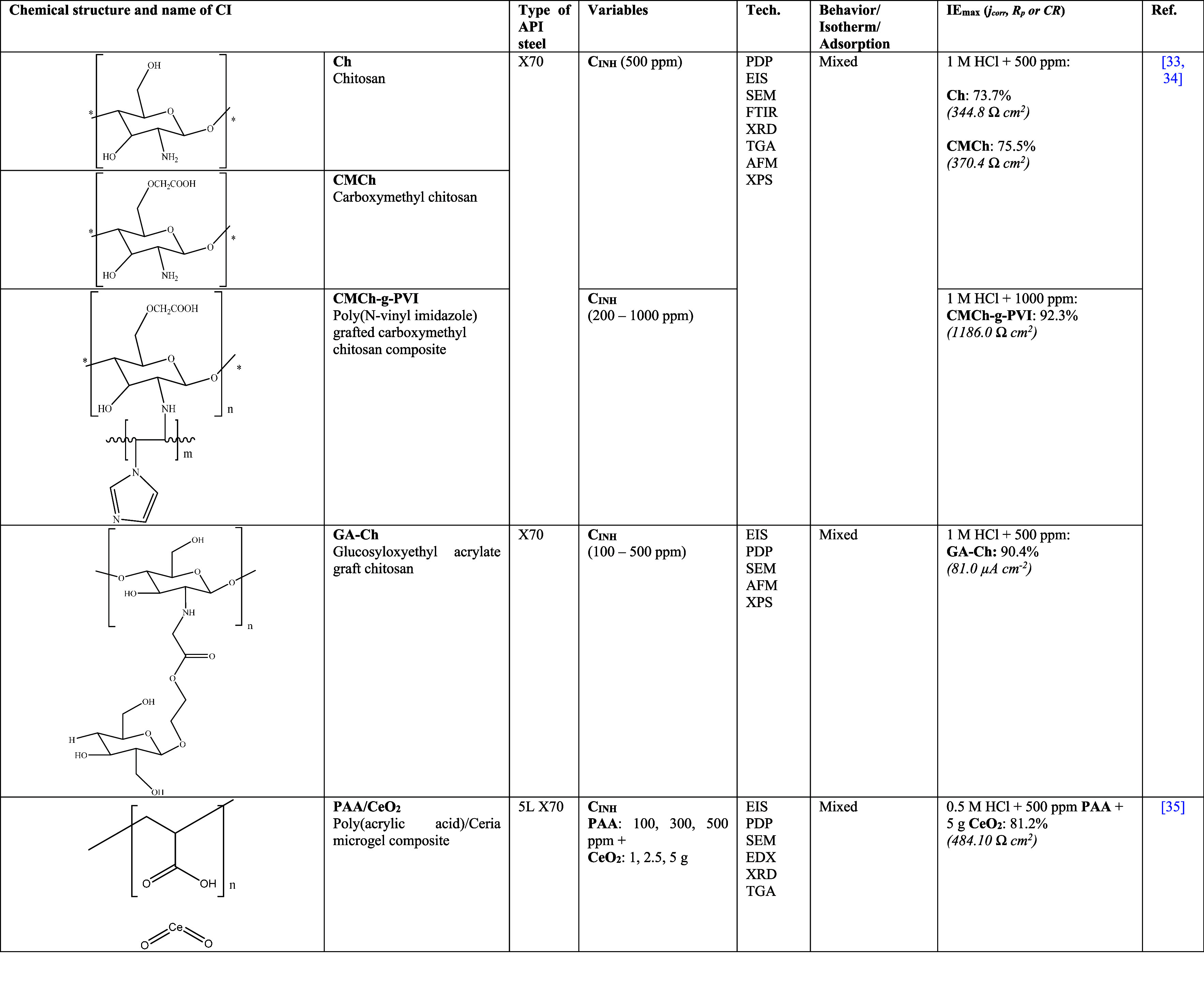
Corrosion Inhibitors of Natural Origin for API Steels in Hydrochloric Acid (HCl)^25,26,30−35^

As for Umoren et al.,^30^ these researchers used *Phoenix dactylifera* leaves as raw matter to synthesize a CI for API 5L X60 immersed in HCl at 15 wt %, where the antioxidant activity of the butanolic extract (BUT) was due to the presence of phenolic acids, flavonols, flavones, and their derivatives.^37^ In order to increase their *IE*, three synergistic agents were employed, formic acid (FA), potassium iodide (KI), and zinc nitrate [Zn(NO_3_)_2_], finding a favorable synergistic effect that increased the *IE* to 96.9%, 94.9%, and 78.22%, respectively. These results were associated with the cooperative adsorption of BUT extract with KI and FA through I^–^ and CO, respectively. In contrast, when a higher amount of Zn(NO_3_)_2_ was added to the BUT extract of the *P. dactylifera* leaves, an antagonistic behavior pattern occurred, which originated from the competition of species for the active sites on the metal surface, thus reducing the *IE*.

Likewise, in 2019, Espinoza-Vázquez et al.^31^ studied the behavior of three commercial carbohydrates (D(+)-glucose, D(+)galactose, and lactose (Table 2), obtaining **IE**_max_ values of 77.9, 74.0, and 89.6% at 50 ppm, severally. Afterward, the research work was extended through the synthesis of new compounds derived from glucopyranose, mannopyranose, and hexahydropyran, and it was concluded that the carbohydrate modification did not exert any significant effect on the *IE* of the compounds, which was equal to 76.4 and 87.5% and attributed to the presence of −OH in molecule rings; these results suggested that the addition of functional groups during the synthesis process led to *IE* diminution. Meanwhile, Sánchez-Eleuterio et al.^32^ evaluated six new carbohydrate–xanthine conjugates linked through a 1,2,3-triazole ring for API 5L X70 steel in 1 M HCl. During the EIS tests, it was observed that the compounds featuring theobromine or theophylline + glucose or galactose (**3b**–**d**, Table 2) presented *IE* > 90% from C_INH_ at 20 ppm. This behavior pattern was confirmed through PDP, and in the case of theophylline + glucose (**3a**), the authors indicated that the factors that reduce the molecule electron contribution are the stereochemistry and spatial arrangement of the **3a** conjugation, which promote a bad orientation of the molecule during the adsorption process, thus limiting the *IE* due to the reduced interaction with the metal surface. For compounds **3b**–**d**, the OAc (−CH_2_–OH) group of the pyranoic ring is possibly the main active center that interacts with the steel surface. The analysis of 3D images obtained by AFM and SEM micrographs confirmed the roughness loss and surface damage in the presence of theobromine + glucose (**3b**) or lactose (**3f**), respectively. In order to have a better interpretation of the results, a deep DFT analysis of the structures, including the whole protonated form, was carried out, finding that none of the CIs presented a planar configuration. In contrast, only the theophylline and theobromine parts preserved a planar configuration associated with the high electron dislocation in resonant systems. Through the MO analysis, it was observed that the HOMO was mainly distributed on the glucose, galactose, and lactose groups, whereas the LUMO, in the case of **3d**–**f**, was on the xanthine groups; for **3a** and **3c**, it extended to the triazole ring, and in the case of **3b** only on the triazole ring. The authors suggested that the molecules with flat structures had a higher probability of interacting by donation/backdonation through OH functional groups. In the past few years, the innovation in the production and synthesis methods has allowed the development of CIs from biopolymers, obtained from natural and renewable sources, and their modification and hybridation toward more complex materials. For example, Bentrah et al.^26^ studied gum arabic (GA) (as a dry powder), oozed by trees of the *Acacia Senegal* species and whose main compound is arabinogalactan, as CI of API 5L X42 steel in 1 M HCl.

The authors attributed its *IE* of 92% to the amphiphilic nature of GA, where the functional groups hydroxyl (−OH) and carboxyl (−COOH) provide the hydrophilic part, whereas the biopolymer proteins represent the hydrophobic part. In addition, it was suggested that the structure can be protonated in the carbonyl groups (C=O), thus forming a polycation that can interact with Cl^–^ ions adsorbed on the surface through physisorption processes. For this reason, the hydrophilic and hydrophobic regions can work together, where in the first region, active anchoring sites are located, and in the second region, the function of isolating the metal surface due to higher occupied volume takes place.

Likewise, in 2018, Eduok et al.^33^ obtained polymers with even more complex chemical structures by performing the graft copolymerization in CIs for API X70 steel in 1 M HCl based on the chitosan biopolymer, adding the carboxymethyl group to give hydrophilicity to the macromolecule. In another study by Hu et al., the grafted polymer poly(vinyl imidazole) was studied in order to synthesize a new hybrid polymeric composite with higher water solubility due to the fact that it is a polymer characterized by its water solubility and easy protonation in acid media.^38^ The authors compared the morphology of the metal surface in the absence and presence of CIs, identifying slight structural modifications (scaly and subfibrous) attributed to the carboxymethylation process of chitosan, whereas the grafting of poly(vinyl imidazole) provoked a more amorphous morphology in a densely packed polymeric network structure as well as an increase in its thermal stability according to TGA studies. In the electrochemical tests, a slight increase in the *IE*_max_ (∼2%) was observed with the first grafting; in contrast, with the second grafting and an increase in the C_INH_, the *IE*_max_ grew ∼17%. Similarly to the previous study, the XPS analyses suggested that the imidazole rings were protonated through the =N^+^H– bond, thus facilitating the adsorption and formation process of an insulating chitosan film on API X70 steel through Fe bonds. Afterward, in 2020, Eduok et al.^34^ carried out another study for API X70 steel in 1 M HCl using grafted glucosyloxyethyl acrylate in order to increase the chitosan hydrophilicity. From these studies, it was concluded that the groups with which chitosan was grafted played a major role in the *IE*, promoting a higher adsorption of macromolecules on the metal surface and occupying a higher surface area. Equally, there was an important effect of the polymeric chains and hydroxyl (−OH) and amine functional groups. In another study carried out in 2017 by the same research group^35^ for API 5L X70 steel in 0.5 M HCl, a water-soluble hybrid composite based on a ceria/poly(acrylic acid) microgel was synthesized. It was found that the addition of ceria (CeO_2_) favored the adsorption process of poly(acrylic acid), which increased the *IE* up to 24% at concentrations of 5 g of CeO_2_ and 500 ppm of poly(acrylic acid). However, with 1.0 and 2.5 g of CeO_2_, a significant effect on the inhibiting activity was not observed, which contradicted what has been reported in the literature that suggests that Ce oxides/hydroxides can reduce the attack of chloride ions (Cl^–^) in acid medium.

However, research works featuring CIs based on organic structures obtained from plant extracts, carbohydrates, biomass, biopolymers, and/or their modifications have not been fully developed probably because there are diverse synthesis processes, which makes the comparison of the obtained compounds difficult, for they depend on the biomaterial origin, technique, and/or solvent to be used. Furthermore, the scarce availability of a thorough and efficient characterization of the active compounds of extracts and carbohydrates limits even more the understanding of the corrosion inhibition mechanism in addition to the high concentrations that have to be employed to reach *IE* > 90% under standard conditions.^30^

On the other hand, also CIs based on drugs,^39−41^ synthetic organic compounds,^23,27−29,42−49^ ionic liquids (ILs),^21,50−55^ and their polymeric derivatives (PILs)^56^ have been studied as shown in Table 3. As for the study of drugs and their active principles as CIs of API steels, it has been limited; notwithstanding, there is evidence of their potential to inhibit corrosion provoked by HCl. Data reported in the literature indicate that the active compounds of drugs consisting, mainly, of combinations of phenols, heterocycles (triazoles, pyran and isoxazole), and compounds with −NH–, −OH, and −SO_2_^–^ provide active sites to their molecules and have resulted efficient (*IE* > 80%) at C_INH_ > 200 ppm under standard temperature conditions and stationary state (298 K and 0 rpm). In addition, for their use at industrial level, it is important that the possible long-term environmental consequences derived from their use such as degradation resistance and growth of antibiotic-resistant microorganisms be considered, in addition to their accidental or on-purpose liberation into the environment.^57−59^ Evaluations of drugs as CIs were carried out by Abeng et al.^39^ and Espinoza-Vázquez et al. in 2020^40^ for API 5L X52 steel in 0.02 and 1 M HCl, severally. Abeng et al. found that heterocycle antibiotics like gentamicin and sulfamethoxazole inhibit acid corrosion with *IE*_max_ values of 92 and 82%, respectively. Through theoretical studies of adsorption isotherms and molecular dynamics simulation with plane wave density functional theory, it was observed that the gentamicin and sulfamethoxazole molecules formed complexes with the metal surface through N, O, and/or S heterocycles, where the gentamicin adsorption energy was higher than that of sulfamethoxazole, where both adsorption mechanisms were physical.

**Table 3:**
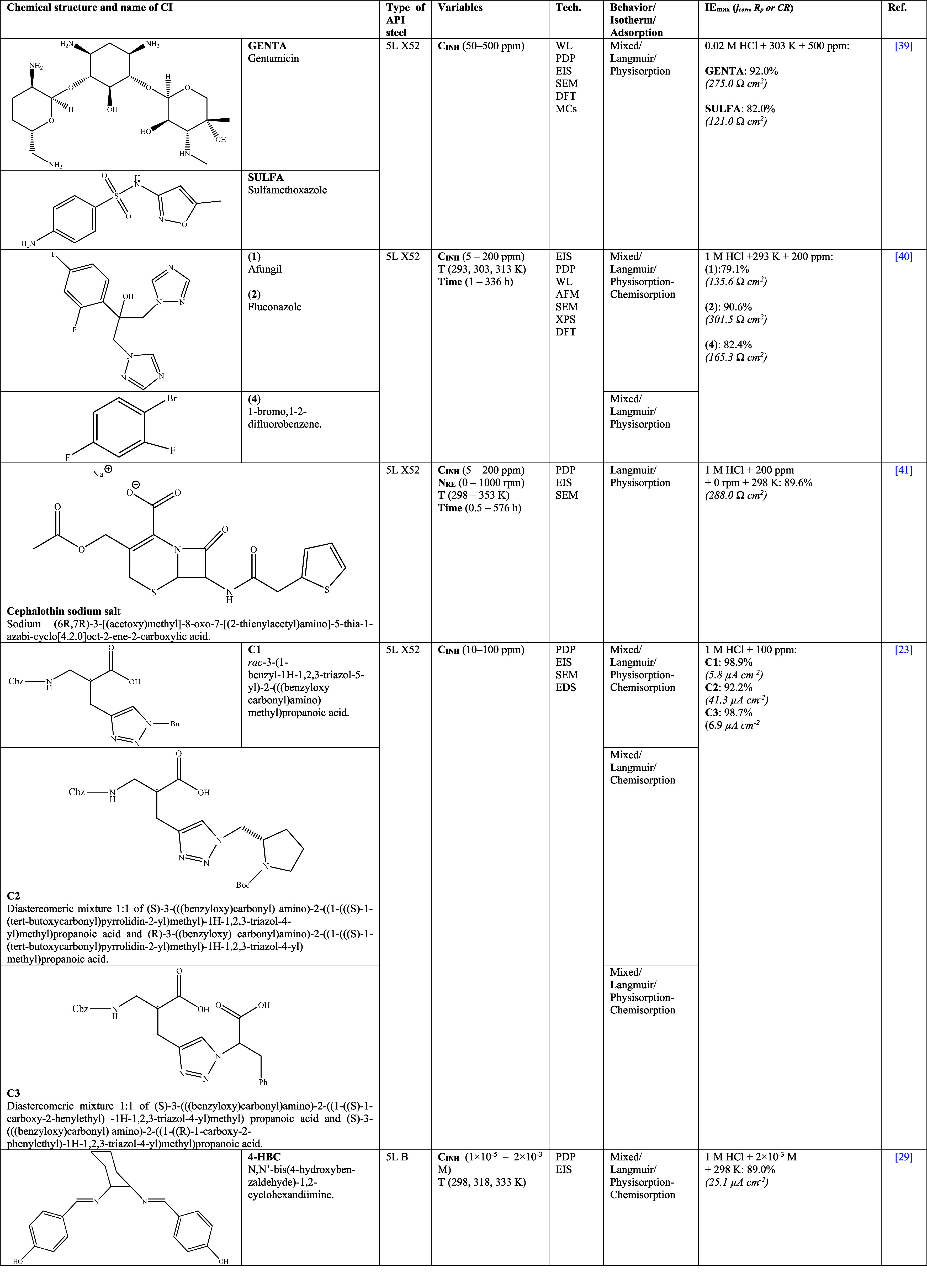

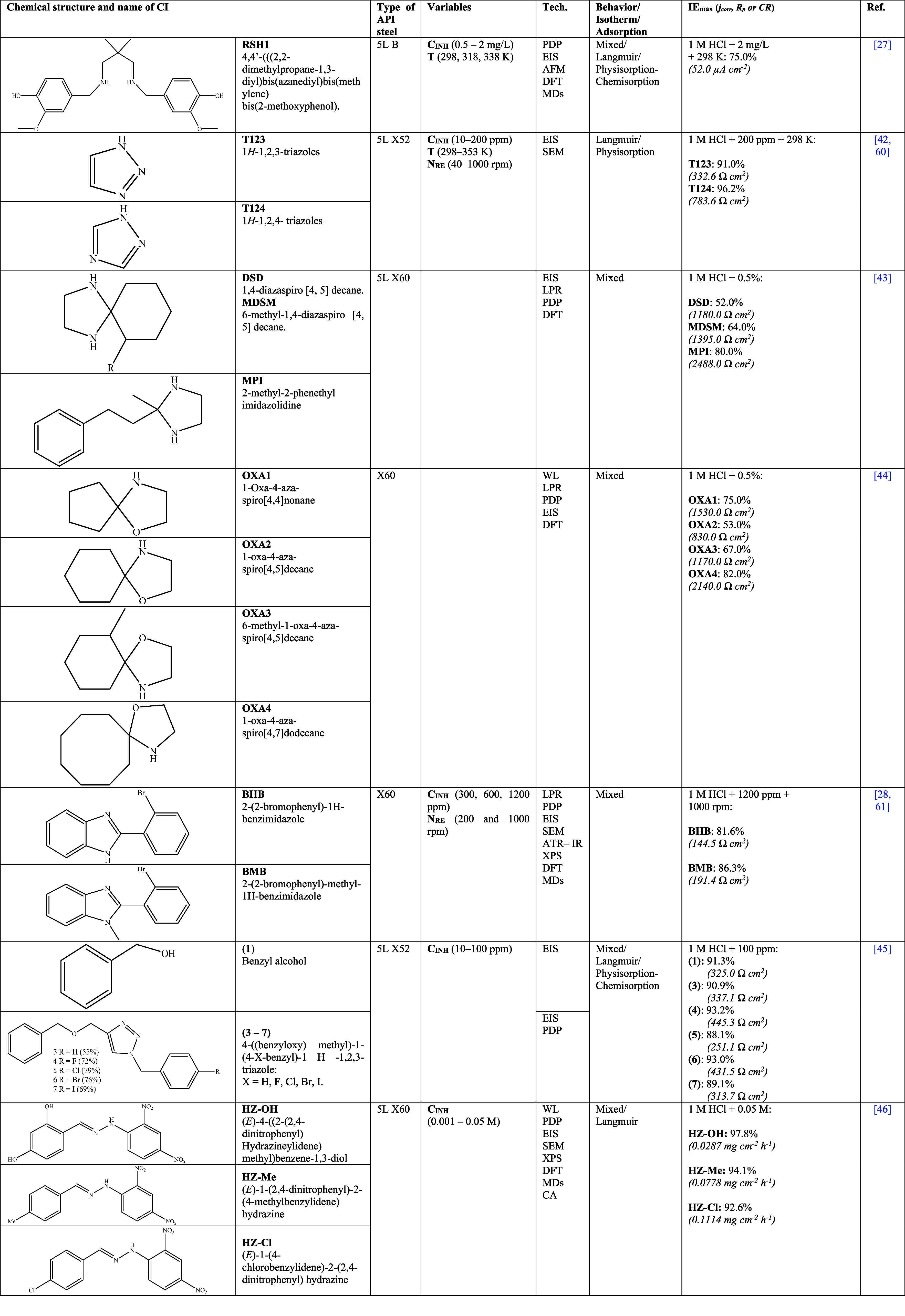

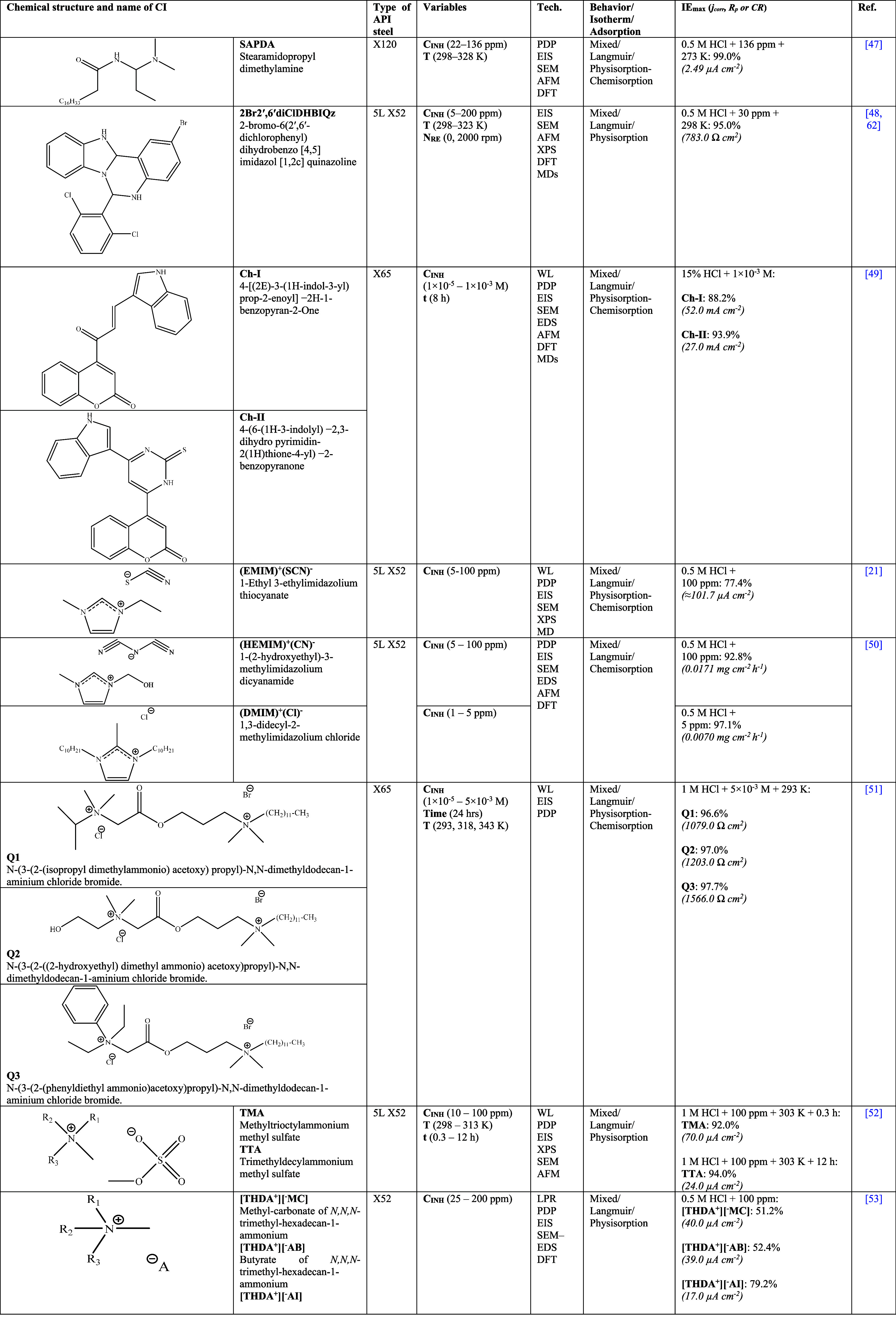

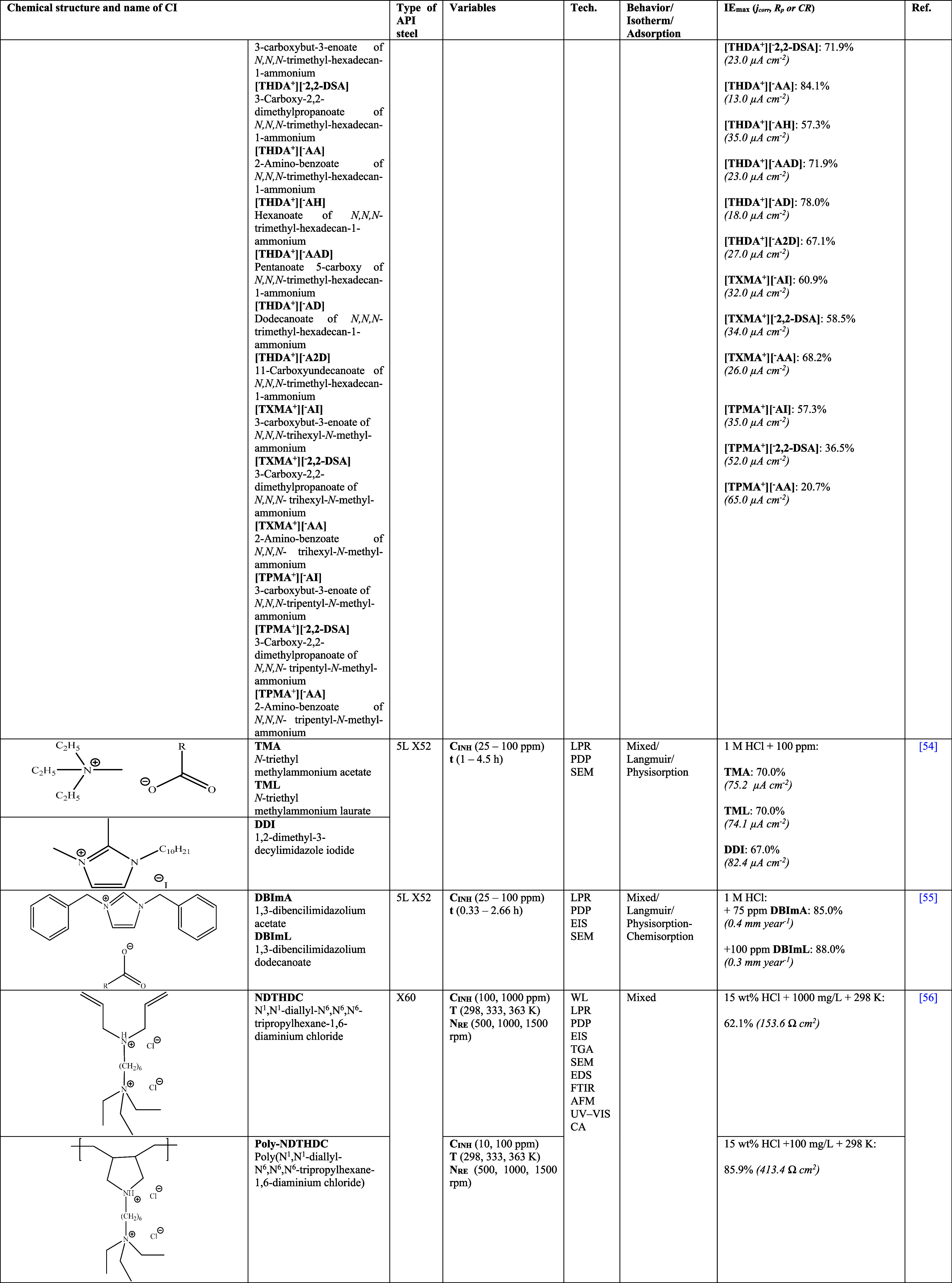
Variety of Synthetic Corrosion Inhibitors for API Steels in Hydrochloric Acid (HCl)^21,23,27−29,39−56,60−62^

As for Espinoza-Vázquez et al., they analyzed the drug Afungil and its active principle and the antifungal fluconazole and its fragments 1,2,4-triazole and 1-bromo-2,4-difluorobenzene. EIS spectra confirmed *IE* values close to 90% in the presence of fluconazole from 5 to 200 ppm, and it was suggested that the active principle presented higher *IE* due to the fact that the enteric presentation of the drugs contains a lower amount of active principle. During the evaluation at different temperatures, it was found that the *IE*_max_ values occurred at 303 K with the formation of a more stable film on API 5L X52 steel at different concentrations. Furthermore, Aldana-González et al.^41^ studied the inhibiting behavior of cephalothin sodium salt for API 5L X52 steel in 1 M HCl; this chemical compound consists of a first generation cephalosporin with a wide range of antibacterial activity and antifungal, antiviral, and antiparasitic effects. The study was performed by varying the C_INH_, N_RE_, and temperature conditions. The authors concluded that this compound was adsorbed on the metal-solution interface through a gradual replacement process of water molecules, which in addition to their size, occupied higher area and blocked active sites with *IE*_max_ up to 89.6% at stationary state and 298 K. Afterward, by modifying N_RE_ to laminar flow, the diameter diminution of the capacitive loops was observed in Nyquist diagrams at C_INH_ < 25 ppm. Likewise, the temperature increase promoted the *IE* reduction, keeping a value of 66% above 313 K, which was attributed to the fact that the cephalothin desorption process was favored. Unlike other works, the authors confirmed that the inhibition process was favored at longer immersion times (576 h) of API 5L X52 steel. The researchers considered that a CI can be adsorbed more efficiently on steel at longer immersion times. In addition, the interactions with adjacent molecules (ions, corrosion products, or the very CI) and/or solvent allow the gradual replacement of water molecules at the metal–solution interface. It is clear that in the different research works on CIs, the effect exerted by substituents on the adsorption mechanism is very important.

On the other hand, Schiff bases are compounds that have attracted much interest as CIs due to their environmentally friendly features and whose synthesis results convenient because they come from comparatively more economical raw materials such as aldehydes and ketones, which are easily available.^63^ Schiff-based compounds as CIs have been studied by different authors like Jafarí et al.^29^ who evaluated *N*,*N*-bis(4-hydroxybenzaldehyde)-1,2-cyclohexandiimine (**4-HBC**) for API 5L B steel in 1 M HCl. The authors suggested that these compounds as CIs were adsorbed through physicochemical interactions, diminishing the heterogeneity of the metal surface by replacing water molecules/aggressive ions at the metal interface and thus blocking the active sites of the steel surface.

Furthermore, it was found that the temperature affected the inhibition process, thus decreasing the *IE*_max_ up to 20% due to a quick adsorption–desorption process of **4-HBC**. Afterward, Jafarí et al.^27^ also studied the corrosion inhibition of API 5L B steel in 1 M HCl in the absence and presence of another Schiff base, employing 4,4′-(((2,2-dimethylpropane-1,3-diyl)bis(azanediyl)bis(methylene)bis(2-methoxyphenol) [**RSH1**]. The authors suggested that this base was adsorbed on the API 5L B steel surface by displacing water molecules by means of its rings and N and O heteroatoms; however, its inhibition capacity diminished significantly through a desorption process with the increasing system temperature. In addition, the structure was analyzed by DFT, and it was found that the HOMO lobes were distributed only toward one of the phenyl rings and O and N heteroatoms, whereas the LUMO was located toward the analogue ring, placing the molecule reactive sites at the structure ends and at a lower extent toward the (C–N–C) center. Through MC, a planar adsorption process on the Fe metal surface was observed, which occurred by means of one of the **RSH1** rings and promoted mainly by the presence of C–O–C and −OH groups.

In order to counter acid corrosion, especially in HCl, also compounds derived from heterocyclic amines have been widely used, which is the case of Espinoza-Vázquez et al.^42^ who in 2017 studied synthetic isomeric triazoles such as 1*H*-1,2,4-triazole (**T124**) and 1*H*-1,2,3-triazole (**T123**) for API 5LX52 steel in 1 M HCl. The *IE* of both CIs revealed dependence on C_INH_ under stationary conditions; in contrast, under flow conditions, it decreased up to ∼30% due to the desorption process by the τ increase. This behavior pattern was observed at different temperatures, producing an *IE* diminution of up to 20%. Additionally, the CI stability was evaluated as a time function, observing that the *IE* was kept constant at 96% after 576 h of immersion, which suggested strong physical adsorption on the metal surface due to the presence of three N atoms and ring aromaticity. The authors ratified the inhibiting effect by SEM analysis, observing changes in the API 5L X52 steel morphology as a result of the reduction of the surface damage. The change of the N position in the structure of the triazole derivatives eased the polarization of the **T123** molecules, increasing the *IE* to 5% and 8% in stationary state at 1000 rpm, which is in contrast with the results obtained with **T124**.

On the other hand, Wazzan et al.^43^ synthesized and studied three imidazoline derivatives as possible CIs of API 5L X60 steel in 1 M HCl: 1,4-diazaspiro [4,5]decane (**DSD**), 6-methyl-1, 4-diazaspiro [4,5]decane (**MDSD**), and 2-methyl-2-phenethyl imidazolidine (**MPI**). By means of electrochemical tests, *IE* values of 52, 60, and 80% were obtained, severally. In the case of **MPI**, the authors associated these results with the presence of electron donor groups such as the terminal benzyl ring and methyl group in the imidazoline ring, which worked as “anchoring” sites in the molecule, unlike **MDSD** and **DSD**, which contained fewer functional groups and displayed less interaction with steel. The DFT analysis of the structures and distribution of the MOs indicated that the reactive sites of the molecule, in the case of HOMO, were mainly distributed over the N atom of the imidazoline rings; as for MPI, it was over the phenyl ring. In the case of LUMO, it was delocalized over the N atom of the imidazoline ring and phenyl ring. The authors emphasize the importance of selecting the substituent group in the C_2_ position of the imidazolines. In order to extend the understanding of the effect of the substituent groups on CIs with heterocyclic compounds, the same authors analyzed four oxazolidines for API X60 steel in 1 M HCl:^44^ 1-oxa-4-azaspiro [4,4]nonane (**OXA1**), 1-oxa-4-azaspiro [4,5]decane (**OXA2**), 6-methyl-1-oxa-4-azaspiro [4,5]decane (**OXA3**), and 1-oxa-4-azaspiro [4,7]dodecane (**OXA4**). In the results of the electrochemical tests, the following *IE* trend was observed: **OXA2** (53%) < **OXA3** (67%) < **OXA1** (75%) < **OXA4** (82%). This behavior pattern was associated with the presence of cyclododecane, which occupied a higher surface area than cyclodecane, and with that of the electron donating group 6-methyl. Afterward, the DFT and MO analyses determined that HOMO was delocalized mainly over the oxazolidine rings with the exception of **OXA3**, which also covered methylcyclodecane. In contrast, LUMO was distributed over the oxazolidine ring and other substituent rings.

In 2020, Onyeachu et al.^28^ also considered organic structures based on heterocycles with N atoms: 2-(2-bromophenyl)-1H-benzimidazole (**BHB**) and 2-(2-bromophenyl)-1methyl-1H-benzimidazole (**BMB**). By means of EIS studies of API X60 steel in 1 M HCl, it was found that by including the methyl group (**BHB** + −CH_3_ → **BMB**), the *IE* increased slightly (∼5%), which was attributed to the fact that the alkyl groups work as electron donors to the ring, thus “activating” it and increasing the electron density through an inductive effect. This behavior pattern was supported by DFT-based calculations, which evidenced that the addition of the methyl group allowed the E_HOMO_ redistribution exclusively over the benzylimidazolium ring. By evaluating **BHB** and **BMB**, a slight decrease in the charge transfer resistance and current density values was observed due to the flow rate change, which indicated that the products of Fe/CI interactions remained adsorbed, i.e., the hydrodynamics did not affect significantly the adsorption process of this type of CIs. In order to complement these results, the ATR-IR technique in the absence and presence of CI was employed, finding the characteristic signals of the CIs and the Fe–O bond, which were the strongest ones for **BMB**. Through SEM analysis of the sample without CI, it was concluded that the surface pitting was attributed to the electromigration process of the Cl^–^ ions; in contrast, with CI, the amount, size, and depth of the pitting diminished because of the inhibiting effect of the protonated imidazole and electrostatic interactions between the CI and Cl^–^ ions adsorbed on the API X60 steel surface. The capacity to displace water molecules displayed by the CIs was confirmed by MCs, and it was found that the adsorption energy of the CIs was higher than that of water.

The use of different halides with benzyl alcohol and triazoles as CIs was reported by Espinoza-Vázquez et al. in 2021 for API 5L X52 steel in 1 M HCl^45^ (Table 3). The authors suggested that halides such as F, Cl, Br, and I work as electron donating elements by providing higher availability of electrons because of having a pair of free electrons, which improves the interaction process with the metal surface of API 5L X52 steel. Additionally, it was suggested that triazoles protonated due to the acid medium, interacting with both the metal and the present Cl^–^ ions. By means of dynamic simulations, it was found that the compounds **1**, **3** (−H, in the absence of halogens) and **5** (−Cl) interacted through the benzyl ring; instead, **4** (−F) presented higher interaction through the N atom of the triazole ring (Table 3). The triazole derivatives presented specific behavior patterns depending on the halide in their structure. In contrast, compound **6** (−Br) was adsorbed through the halogenated ring. The authors did not observe any significant change in *IE*_max_ as a function of the substituted halide, which was in contrast with benzyl alcohol in their absence. Similar conclusions were obtained in 2018 by the same research group^23^ in a previous study on racemic and diasteromeric mixtures of triazoles derived from β-amino acids for the same API steel type and medium, where the effect of the immersion time on the metal samples was analyzed, finding that the CI was effective up to 336 h with an *IE* of 85.7%; afterward, a desorption process occurred, which diminished the *IE* up to 36.2% after an immersion time of 504 h.

Khamaysa et al.^46^ studied structures based on hydrazone (Table 3) for API 5L X60 steel in 1 M HCl. These compounds are considered as nontoxic and with inhibiting activity due to the presence of the azomethine active group (−NHN=CH−), which possesses electrophilic and nucleophilic character, as well as π electrons. In the study, three structures with different functional groups were employed: 1,3-diol (−OH), 4-methyl (−Me), and 4-chlorine (−Cl), which provided nonbonding electrons and molecular electronegativity differences. In WL and electrochemical tests, important changes in the inhibiting behavior with the change of the functional group were observed. Additionally, by SEM and XPS, the reduction of surface damage caused by physicochemical interactions between the CI and interface was confirmed, which blocked the reactive sites of the API 5L X60 steel. From the CA characterization technique, the increase in the surface hydrophobicity by inhibiting the film was confirmed. In order to support these results, DFT and MD calculations were carried out; with the first ones, the interaction between the molecule and an Fe cluster was observed, finding that the evaluated CIs tended to form covalent bonds with Fe, where the −OH group showed better results than the others because of its electron-donating capacity that reinforced the electron transfer between the CI and Fe. Likewise, it was confirmed that the molecule presented the HOMO through its thorough structure; in contrast, the LUMO was exclusively located over the dinitrophenyl ring. On the other hand, the MD simulation helped confirm the strong planar adsorption of the molecule on the hematite surface through the reactive N and C atoms. Bahgat-Radwan et al.^47^ proposed a corrosion inhibition mechanism featuring stearamidopropyl dimethylamine for API X120 steel in 0.5 M HCl. From their corrosion studies by PDP, EIS, and optical profilometry, it was stated that the CI adsorption occurred through the molecule polar group, located in the N and O heteroatoms, as confirmed by the analysis of molecular orbitals and Mulliken charges by molecular simulation.

Other types of heterocyclic compounds, such as quinazoline and benzopyranones/indole, have been considered as CIs by different authors. Aldana-González et al.^48^ performed the analysis of the inhibiting properties of 2-bromo-6(2′,6′-dichlorophenyl)dihydrobenzo [4,5]imidazo [1,2c]quinazoline for API 5L X52 steel in 0.5 M HCl by EIS, finding *IE*_max_ of 94% from 15 ppm and suggested that the CI adsorption prevented the Faradaic processes that take place on the metal surface. Afterward, the study was extended independently with respect to the temperature and flow, where the temperature increase favored the molecule desorption process. Likewise, with the flow increase, specifically from stationary to transitory, the *IE* decrease occurred by the CI partial desorption. However, the XPS analysis confirmed the dissociation of the molecule in Br^–^ and the cationic form of this CI. This molecule was also analyzed by DFT in its original structure and protonated form, the latter being the one that displayed higher donor–acceptor behavior. Both compounds presented a planar arrangement with rotation of the 1,3-dichlorobenzyl group, as suggested by the DFT and MDs, as well as the formation of new resonant bonds in the quinazoline and imidazole and double bonds at the site, where the Br ion was dissociated. According to the molecular orbitals, quinazoline, imidazole, and benzene rings worked as HOMO and LUMO in *INH*, whereas for *INH*^+^, LUMO was distributed over the 1,3-dichlorobenzyl ring.

On the other hand, Hashem et al.^49^ analyzed API X65 steel in HCl (15%) in the absence and presence of two heterocycles based on benzopyrans and indole bound through an *α*,*β*-unsaturated [3-methylbuten-2-one] carbonyl group and pyrimidine-2(1*H*)-thione. According to data from electrochemical tests, *IE*_max_ values of 88.2 and 93.9% were obtained, severally; likewise, with the WL technique, *IE*_max_ values of 87.7 and 94.2% were calculated, respectively. The inhibiting behavior of these compounds was attributed to the conjugated aromatic rings and presence of N, S, and O heteroatoms; however, the pyrimidine-2(1*H*)-thione heterocycle group increased the electron density of the conjugation delocalized electron and favored the creation of new molecule adsorption centers through the sulfur and nitrogen atoms (=N–, =S, and −NH−).

This behavior pattern was corroborated by DFT and MDs analyses, where the study of molecular orbitals confirmed that the indole group and pyrimidine-2(1*H*)-thione were located in the HOMO, whereas the LUMO was located over 3-methylbuten-2-one/benzopyran-2-one and pyrimidine-2(1H)-thione/benzopyran-2-one. Similar to the quinazoline behavior,^48^ the two heterocycles in this study presented planar adsorption on the metal interface. Different works have evaluated ILs as CIs of API steel due to outstanding properties such as high design versatility that stems from their combination capacity with different counterion types, which favors the synthesis of a big number of CIs.^64^ The use of ILs with nitrogenated cations such as ammonium^51−54,56^ and imidazolium^21,50,54,55^ has been widely studied. Ammonium-based ILs have their ammonium ion hydrogen atoms substituted by (R_4_N^+^) alkyl groups, where the latter can be the same or different. The influence of different substituents on ILs was reported by Hegazy et al.^51^ who carried out different syntheses to obtain new-generation-gemini-type surfactant structures for API X65 steel in 1 M HCl based on quaternary diammonium salts bound by a fatty alkyl chain as nonpolar group (acetoxy group), two hydrophilic groups, and two hydrophobic ones with ammonium halides (Br^–^ and Cl^–^). The difference between each structure stemmed from the functionalization of the closest ammonium to the acetoxy group and Cl^–^ anion, employing the substituents 3-(2-i-propyl-dimethyl), 3-(2-((2-hydroxyethyl))), and 3-(2-(phenyldiethyl)). The analysis of the *IE* with C_INH_ of 1 × 10^–5^, 5 × 10^–5^, and 1 × 10^–4^ M helped observe the effect of the functional group on its inhibiting behavior and the donor–acceptor interaction by the benzyl group was suggested, which provided higher electron availability than the −OH or i-propyl group, thus increasing the *IE* in 14%. In addition to the already known characteristics of the ammonium cations, the authors suggested that the adsorption processes also depended on the donating behavior of the functional group, e.g., the benzyl group possesses three double bonds, pairs of lone electrons, hydrophobicity, and planarity; the −OH group has moderate activation due to its lower donating character, and the i-propyl group does not have any donating character. In the proposed inhibition mechanism, it was suggested that the surfactant positive and negative charges interact through electrostatic attractions with the charges corresponding to the metal surface, including the Cl^–^ ions from the corrosive medium. Another study based on an IL with quaternary diammonium was performed by Odewunmi et al.^56^ for API X60 steel in HCl (15 wt.%), comparing ammonium-chloride derivatives in their vinyl and polymeric forms at different C_INH_ values, temperatures, and flow rates. By comparison of the different *IE*s, it was observed that the polymeric form surpassed the vinyl one, which was associated with the higher number of active sites in a single macromolecule, thus favoring its adsorption on the metal surface before the corrosive medium. It was observed that the *IE* increased at growing temperatures from 298 to 333 K, which was attributed to chemisorption processes with the metal surface and to the thermal stability of the CIs; in contrast, at 363 K, a kinetic energy increase in the corrosive ions took place, provoking the winding down of the *IE*. As for the flow rate change (500 → 1000 rpm), it increased slightly the *IE*, which was related to the more intense transport of inhibiting molecules from the solution toward the steel surface; on the contrary, with the transition from 1000 to 1500 rpm, the removal of inhibiting species from the metal–solution interface and formation of oxides due to the presence of O_2_ occurred. The inhibition mechanism suggested that a mixed-type adsorption process happened with the preferential adsorption of the Cl^–^ anions, creating a surface with an excess of electrons, which allowed the electrostatic interaction of the cationic species, whereas the N heteroatoms were protonated and promoted the formation of coordination bonds and back-donation with the Fe empty d-orbital. On the other hand, in 2018, Arellanes-Lozada et al.^52^ studied ILs based on the ammonium cation and methyl sulfate anion for API X52 steel in 1 M HCl. The authors suggested that the adsorption of this type of CIs on the metal surface took place on the cation hydrophilic part through its N heteroatoms, limiting the charge transfer of the metal surface by means of a geometrical blockage of the steel active sites whose effect increased as a C_INH_ function, whereas the hydrophobic part, i.e., the alkyl chains, was oriented toward the solution core, forming a barrier that prevented the diffusion of corrosive ions. Furthermore, it was suggested that the IL can also group on a second cation layer originated by the interaction between alkyl chains, which prompted the reorientation of the polar group, the N atom of the ammonium cation, toward the solution core. Another research work on organic anions was carried out by Cornejo-Robles et al.^53^ who studied 15 different combinations of ILs based on carboxylate anions and quaternary ammonium for API X52 steel in 0.5 M HCl. By means of electrochemical techniques, the authors suggested that the *IE* is a variable dependent on the anion type as well as on the characteristics of the alkyl chains in the different cations, where CIs with higher presence of alkyl groups in short alkyl chains presented better *IE*. Likewise, it was reported that anions such as 3-carboxybut-3-enoate, dodecanoate, and 2-amino-benzoate, in combination with the cation trimethyl-hexadecan-1-ammonium, displayed enhanced behavior as CIs in comparison with other cation/anion combinations in evaluated ILs. Finally, by DFT analysis, it was confirmed that the carboxylic groups (COO^–^) and amine/ammonium (NH_2_/N^+^–C_4_) were the most reactive regions of the most efficient ILs. In contrast, in 2014, Olivares-Xometl et al.^54^ studied ILs with the cation triethyl-methylammonium and organic anions (acetate and laurate) as CIs for API 5L X52 steel in 1 M HCl. The obtained *IE* results did not show significant differences with the anion change. These ILs were analyzed with respect to the IL consisting of an imidazolium ring as cation and iodide as anion, considering that halides are widely used synergistic agents due to their good performance in combination with organic compounds, especially with those that possess π electrons such as heterocycle cations.^65−67^ The authors concluded that ILs combining a quaternary ammonium as cation and an organic anion with carboxylic groups displayed similar *IE*s (∼70%) to those of ILs with imidazolium as cation and a halide as anion.^54^

In the study carried out by Lozano et al.,^55^ it was reported that by incorporating an aromatic ring (benzyl) into the imidazolium cation of the ILs with organic anions derived from carboxylic acids (acetate and dodecanoate), the *IE* was between 85–88% inhibiting the corrosion of API 5L X52 steel in 1 M HCl. This result was mainly attributed to the functionalization of the terminal alkyl groups of the imidazolium ring with benzyl groups, which contributed π electrons to the cation, thus favoring the orientation of the CI toward the metal surface. Likewise, in 2019, Corrales-Luna et al.^21,50^ evaluated the behavior of imidazolium ILs with three different anions for API 5L X52 steel in 1 M HCl: 1-ethyl 3-methylimidazolium thiocyanate (*IE*_max_ = 77.4%), 1-(2-hydroxyethyl)-3-methylimidazolium dicyanamide (*IE*_max_ = 92.8%), and 1,3-didecyl-2-methylimidazolium chloride (*IE*_max_ = 97.1%). The authors concluded that the changes in the Tafel curves were attributed to reorientation processes of the rings in order for them to be adsorbed on the metal surface. In addition, it was suggested that the imidazolium ring cations, independent of the alkyl chain length or presence of functional groups, interacted with API 5L X52 steel through electrostatic interactions with negatively charged ions (aggressive anions and those of the CI). In contrast, the anionic part of the ILs competes for the active sites to displace water molecules and/or Cl^–^ ions, which in turn allows the attraction of its counterion. In the case of 1,3-didecyl-2-methylimidazolium chloride, the longest alkyl chains covered a higher surface area on the API 5L X52 steel, which explains its high *IE* with respect to other ILs. Finally, the authors analyzed the temperature effect on the inhibiting behavior at C_INH_ from 1 to 5 ppm of 1-(2-hydroxyethyl)-3-methylimidazolium dicyanamide and 1,3-didecyl-2-methylimidazolium chloride; in both cases, a significant trend in the *IE* behavior was not found, which was attributed to the complexity of the interfacial phenomena that can occur. Research works on CIs evaluated in HCl media have shown that despite great efforts to diminish the diffusion and attack of Cl^–^ ions on the API steel surface, most CIs require C_INH_ above 100 ppm. This is due to the fact that HCl is a strong acid with a high acid dissociation constant (*K*_a_ = 1.3 × 10^6^) that generates a high concentration of protons in the corrosive medium that accelerates the reaction rate of the cathodic reactions and corrosion of steel. For this reason, the CI desorption process from the metal surface is unavoidable and is favored by intermolecular movements that occur because of the increase in the temperature and system flow conditions.

#### 3.1.2 Corrosion Inhibition of Sulfuric Acid Environment

Sulfuric acid (H_2_SO_4_) is one of the most produced reagents in the world and with industrial applications that are similar to those of HCl;^68^ additionally, it is usually used as catalyst in oil processes, pigment production, steel treatment, extraction of nonferrous metals, explosive production, detergents, plastics and fibers.^69^ Especially for the oil industry, the concern has grown due to its corrosive effect on tanks and pipes due to the transformation of H_2_S and SO_X_, generated during oil extraction and refining, into concentrated H_2_SO_4_. Since this is a strong acid, it requires safe storage and transport conditions because as in most corrosion mechanisms, its presence promotes the immediate metal attack, generating the formation of hydrogen gas and ferrous ions. The corrosion process of steel by H_2_SO_4_ generates iron sulfate (FeSO_4_) (Reaction 4) as the main corrosion product, where the corrosion rate depends mainly on the oxidant diffusion (H_2_SO_4_) toward the steel surface:^70^
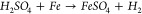
4

Table 4 shows CIs that have been proposed to inhibit the corrosion of API steels by H_2_SO_4_. In the past decade, the number of studies focused on employing plants or drugs in H_2_SO_4_ corrosive media has been scarce due to the low obtained *IE*s. However, an interesting proposal was done by Golchinvafa et al.^71^ to employ *Fumaria officinalis* extract for API X80 steel in 1 M H_2_SO_4_. This species has a wide variety of chemical organic compounds that are relatively harmless and of natural origin like potassium salts, fumaric acid, flavones, flavonols, flavanones, flavan-3-ols, anthocyanins, dihydroflavonols, and isoflavones, glycosylated or acylated derivatives, and oligomeric and polymeric structures, etc.^72^ The authors suggested that their inhibiting behavior depended on the concentration of the H_2_SO_4_ oxidant agents as well as on the functional groups of the chemical compounds present in the *Fumaria officinalis* extract that work as anticorrosive agents located in the metal interface. Furthermore, it was reported that the *IE* displayed an inverse behavior pattern with respect to temperature, which was related to the reduction of the physical interaction between chemical species present in the extract and steel surface, e.g., if the temperature increased up to 333 K even with C_INH_ of 6 g/L, *IE*_max_ diminished at least by 33% as a consequence of a higher CI desorption rate. From the SEM–EDS analysis, the authors attributed the surface irregular sections to the formation of iron sulfides (FeS) and not to characteristic corrosion products formed with the SO_4_^2–^ ion. On the other hand, Flores-Cortez et al.^2^ evaluated metronidazole [2-(2-methyl-5-nitro-1H-imidazol-1-yl)ethanol] for API 5L X52 steel in 1 M H_2_SO_4_. This chemical compound is a drug belonging to the nitroimidazole group that presents antibiotic and antiparasitic properties and that is obtained by purifying its commercial form Flagyl 500 mg. It was found that the inhibition process with metronidazole presented a C_INH_ of 1.2 mM/L and *IE* of 39%, which increased to 72% when C_INH_ was diminished to 0.6 mM/L; the authors suggested that this behavior pattern was due to the diminution of kinetic activity in the adsorption process.

**Table 4:**
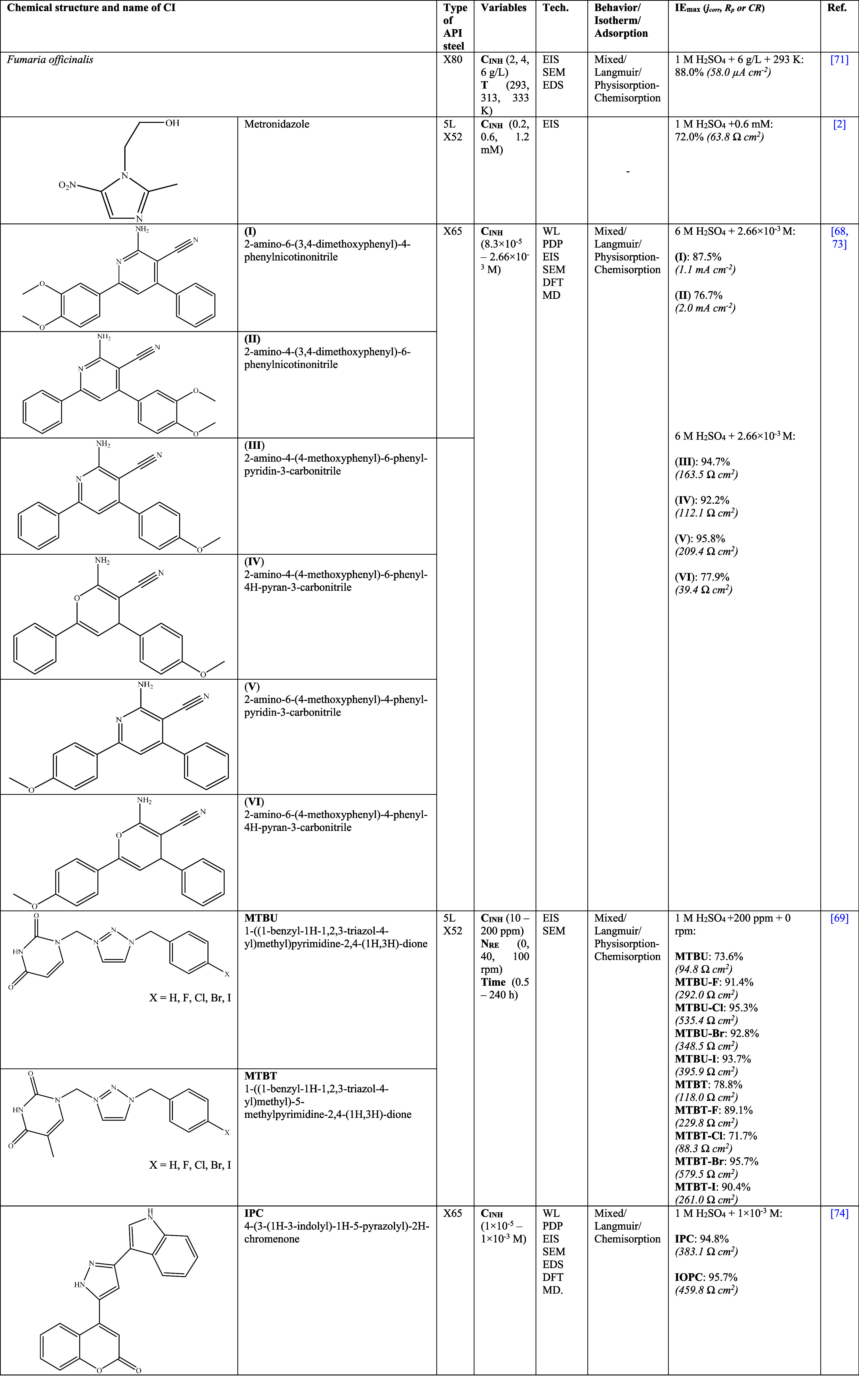

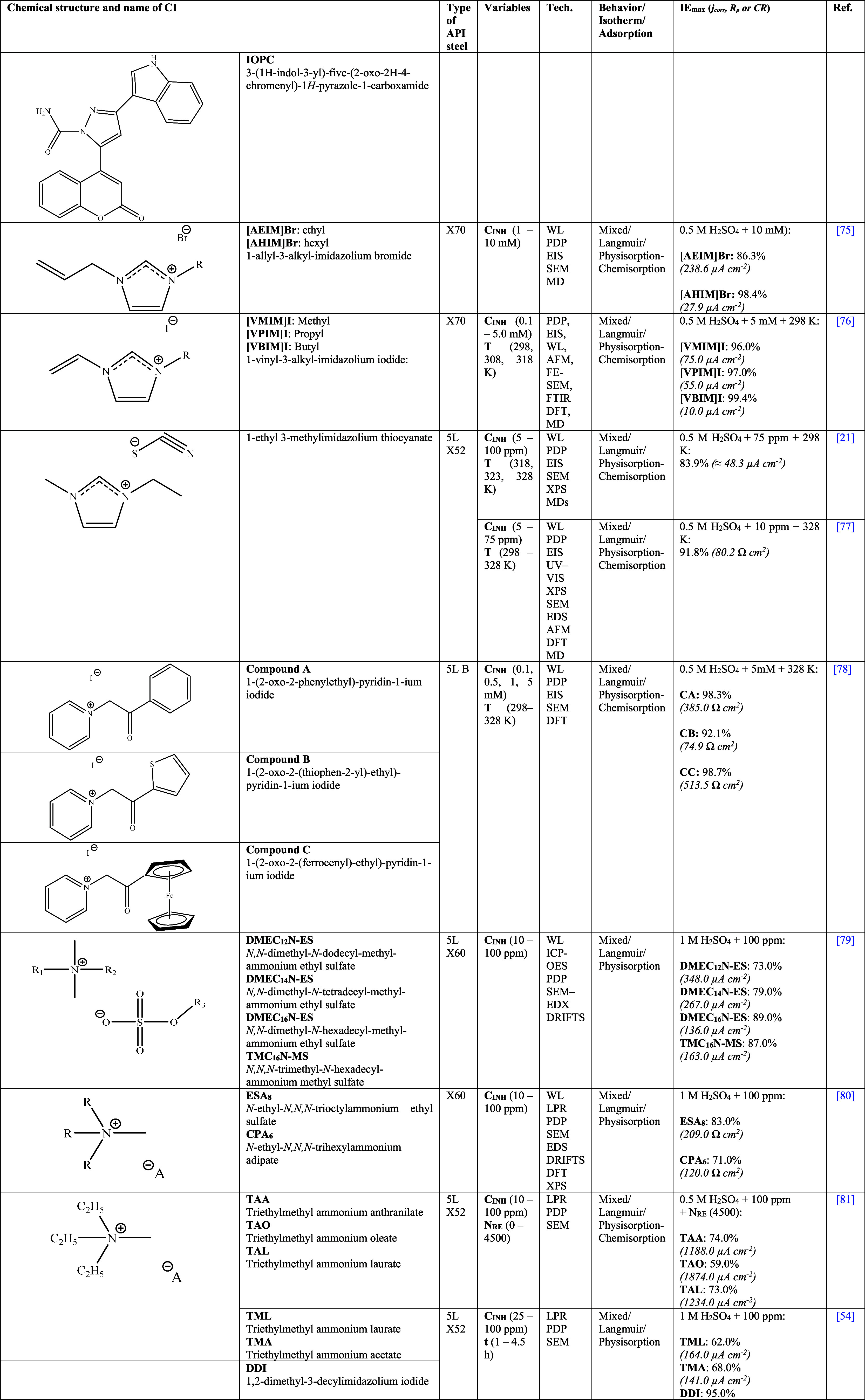

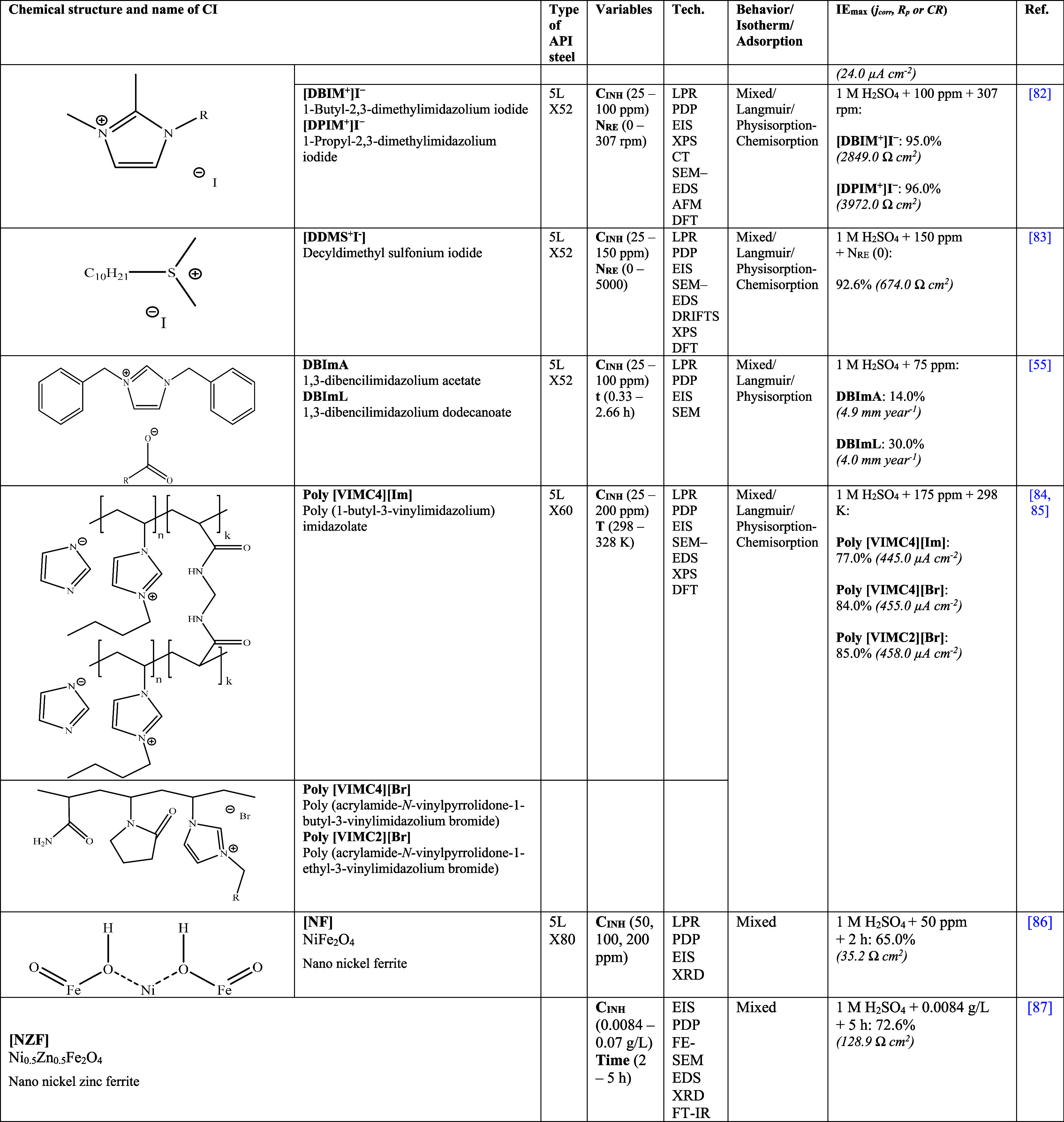
Miscellaneous Corrosion Inhibitors for API Steels in Sulfuric Acid (H_2_SO_4_)^2,21,54,55,68,69,71,73−87^

In this area of research works, studies on the synthesis of organic CIs are fundamental, which have shown a preference for nitrogenated compounds such as pyran and/or pyridine,^68,73^ triazole/pyrimidine^69^ and chalcone^74^ groups. The authors Farag et al.^68^ and Anwer et al.^73^ evaluated 6 similar structures, 4 based on pyridine and 2 on pyran in an extremely acid medium of 6 M H_2_SO_4_ for API X65 steel. As observed in Table 4, the difference between both works was the addition of the methoxy group, the exchange of the pyridine ring by pyran, and the position of the n-methoxyphenyl group. The addition of these CIs to the H_2_SO_4_ medium did not modify significantly the corrosion mechanism, forming a protective film by replacing the water molecules, SO_4_^2–^ and H^+^, thus diminishing the charge transfer from the metal surface to the solution core, exhibiting *IE*_max_ results from 77 to 96%. The reduction of the *IE*s of 2-amino-4-(3,4-dimethoxyphenyl)-6-phenylnicotinonitrile with respect to 2-amino-6-(3,4-dimethoxyphenyl)-4-phenylnicotinonitrile was attributed to steric hindrance effects caused by the closeness of the methoxy (−OCH_3_) and nitrile (−C≡N) groups. A similar behavior pattern was reported for the CIs 2-amino-6-(4-methoxyphenyl)-4-phenyl-pyridin-3-carbonitrile and 2-amino-4-(4-methoxyphenyl)-6-phenyl-pyridin-3-carbonitrile, where 2-amino-4-(4-methoxyphenyl)-6-phenyl-pyridin-3-carbonitrile displayed less steric hindrance due to the presence of just one methoxy group. In contrast, pyrans showed higher *IE* when the methoxy group was closer to nitrile, which occurred due to the redistribution of electrons shared with the pyran group. In general, the pyridine groups displayed better *IE* than pyrans (**V** > **III** > **IV** > **I** > **VI** > **II**, see Table 4) because such behavior pattern was associated with the methoxy groups and the high conjugation of N with the benzene rings, which produced a dense electron cloud that facilitated the electron transfer from the functional group to Fe on the metal surface, thus favoring the formation of coordinated bonds. From the DFT analysis, it was observed that the HOMO lobes were distributed over the 3,4-dimethoxyphenyl and 4-methoxyphenyl groups.

Likewise, the LUMO was distributed throughout the whole molecule in both cases. This behavior was related to the high similarity and amount/type of present functional groups; additionally, the MD simulation confirmed the planar adsorption of the CIs. The authors distinguished three stages in the inhibition mechanism, which were based on possible interactions with the heteroatom or functional group of the CI molecule. At stage 1), physical adsorption through the electrostatic interaction between protonated pyridine and previously adsorbed SO_4_^2–^ ions was considered; at stage 2), the chemisorption of N and O heteroatoms (nonshared electron pairs) and aromatic rings (delocalized π electrons) with the vacant iron d-orbital was suggested, and finally, at stage 3), backdonation by means of Fe d-orbital electrons with the pyridine vacant antibonding orbital was considered.

On the other hand, in 2018, Espinoza-Vázquez et al.^69^ had the hypothesis that nucleic acids such as uracil and thymine, based on pyrimidines, can be efficient CIs for API 5L X52 steel in 1 M H_2_SO_4_, reporting *IE* ≈ 95.3%, where thymine was more efficient than uracil. Furthermore, it was indicated that the addition of halogens (F, Cl, Br, and I) to the organic structures of triazoles did not show a typical *IE* behavior pattern even when in the literature it has been reported that these compounds have played a major role in inhibition processes due to characteristics such as ionic radius and electronegativity. Likewise, such inhibitors were evaluated under hydrodynamic and immersion time conditions, observing the resistance to shear stress in laminar flow (40 and 100 rpm) of the inhibiting film formed by 1-((1-benzyl-1H-1,2,3-triazol-4-yl)methyl)pyrimidine-2,4-(1H,3H)-dione (**MTBU-I**) and 1-((1-benzyl-1H-1,2,3-triazol-4-yl)methyl)-5-methylpyrimidine-2,4-(1H,3H)-dione (**MTBT-Br**). In the first case, the *IE*_max_ values remained at 90% for both flow rates due to fast adsorption/desorption processes that allowed to keep stable the CI adsorbed on the surface. In contrast, when the corrosion rate increased, the **MTBT-Br***IE* also grew slightly to ∼3%, which was attributed to the presence of Br and the thymine methyl group. With respect to the evaluation for long time periods, the resistance to metal corrosion diminished because of the damage of the CI protecting film.

As for Mohamed et al.,^74^ these researchers evaluated API X65 steel in 1 M H_2_SO_4_ in the presence and absence of two chalcone derivatives, which were α,β-unsaturated carbonyl groups, that are found in natural and synthetic products with antibacterial, antifungal, and antioxidant activity. As a result of the PDP and EIS electrochemical tests, it was observed that at C_INH_ of 1 × 10^–5^ and 5 × 10^–5^ M there was an *IE* difference of at least 11% due to the addition of the formamide group (NH_2_C=O) that worked as an electron donating group in the 3-(1H-indol-3-yl)-five-(2-oxo-2H-4-chromenyl)-1H-pyrazole-1-carboxamide (**IOPC**) structure, i.e., it was an anchoring or linking point for steel. From this concentration, the *IE* values tended to reach the “equilibrium” with minimal differences as C_INH_ increased. Like the DFT calculations, the MO distribution presented the same behavior pattern in both molecules, indicating that HOMO was located over the indole and pyrazole groups; in the **IOPC** case, this one was found over the formamide (–HN–C=O). In contrast, the LUMO was distributed through the 2H-chromen-2-one group, pyrazole, and also the formamide (–HN–C=O). This analysis confirmed that the main active center in both molecules was the pyrazole ring. Additionally, the Mulliken charges confirmed that both CIs presented an excess of negative charge around the heteroatoms and some C atoms that contain delocalized atoms, which facilitated the interaction with Fe of the metal surface.

Notwithstanding, research on ILs as CIs is very relevant for H_2_SO_4_ media, where structures based on imidazolium,^21,54,55,75−77,82^ pyridinium,^78^ ammonium,^54,79−81^ and sulfonium^83^ are frequently employed. It has been confirmed that CIs with heterocycle cations, mainly ILs with the imidazolium ring, counter the corrosive effects of API steels in H_2_SO_4_ at different concentrations. In 2020, Arellanes-Lozada et al.^82^ found that the combination of ILs substituted with short chains such as 1-butyl-2,3-dimethylimidazolium and 1-propyl-2,3-dimethylimidazolium, with iodide anions, produced highly efficient CIs (*IE* ≈ 95%) for API 5L X52 steel in 1 M H_2_SO_4_. In addition, another study carried out by Olivares-Xometl et al.^54^ suggested that imidazolium ILs with short chains and a longer chain at position N3, like 1,2-dimethyl-3-decylimidazolium in combination with iodide, also allowed the achievement of similar *IE* for the same steel type and medium. The former studies confirmed the synergistic characteristic of halides by behaving as a kind of bridge between the surface and IL as a result of the interactions of ionic pairs between the organic cation and halide anion, thus contributing to a higher degree of covered metal surface. According to the foregoing, the inhibiting effect of halides has, in general, the order I^–^ > Br^–^ > Cl^–^, which facilitates both the IL adsorption through the creation of molecule dipoles that promote an improved orientation toward the metal surface and the subsequent adsorption of IL cations.^88^ Likewise, the authors emphasize the influence of the length and position of the alkyl chains on the inhibiting capacity of the imidazolium ring. Similarly, Lozano et al.^55^ found that ILs with substituents such as aromatic rings at N1 and N3 positions of the imidazolium rings, like 1,3-dibenzylimidazolium, did not exert any inhibiting effect on a poorly protected API 5L X52 steel surface (*IE* < 30%) in 1 M H_2_SO_4_. This behavior pattern showed that even the presence of organic anions with carboxylate groups like acetate and dodecanoate and the symmetry of the imidazolium cation with benzyl alkyl chains are not enough to achieve a functional combination as CI due to the formation of iron oxides and/or iron hydroxide, which limit the IL adsorption.

In the studies by Zhang et al.^75^ and Feng et al.,^76^ the effect of the alkyl chain on the inhibition process of API X70 steel exerted by an imidazolium IL in 0.5 M H_2_SO_4_ was analyzed. In the first study,^75^ the structures of 1-allyl-3-alkyl-imidazolium bromide with ethyl (**[AEIM]Br**) and hexyl (**[AHIM]Br**) chains (see Table 4) were investigated by PDP and EIS. It was found that the *IE* varied from 6 to 16% when comparing the two CIs under the same conditions, being **[AHIM]Br** slightly better than **[AEIM]Br**; such behavior was attributed to the increased hydrophobic character given by the increase in the alkyl chain.

This study was complemented with MD whose results suggested that both ILs were adsorbed parallel to the Fe(110) surface. In the second study, carried out by Feng et al.,^76^ structures based on 1-vinyl-3-alkyl-imidazolium iodide with three different alkyl chains were evaluated: **[VMIM]I**: methyl, **[VPIM]I**: propyl, and **[VBIM]I**: butyl (see Table 4). The structures with longer alkyl chains displayed improved inhibition behavior at low concentrations (increase of ∼5%), and the authors suggested that the alkyl chains participate as electron donor agents. Afterward, the WL tests at 4 h and different temperatures showed the same behavior, with respect to C_INH_. However, the *IE*s revealed two behavior patterns with the temperature: 1) at low C_INH_, the *IE* diminished up to 17%, i.e., the desorption of the CIs was facilitated, and 2) at high concentrations, the ILs were stable at the evaluated temperatures (Δ*IE* ∼ 1%). Also, theoretical calculations were employed to support the results obtained during the electrochemical tests and surface analyses; nevertheless, an MD simulation was performed only considering the IL cationic part. The results suggested that the adsorption process of the ILs occurred through two main stages: the adsorption of I^–^ on the metal surface and then the adsorption of the imidazolium ring by means of electrostatic interactions. However, it has to be considered that the ILs with halogen anions are being prohibited as CIs due to the environmental problems that result from their synthesis.^89^

In contrast, in 2019, Corrales-Luna et al.^21,77^ published two studies on an imidazolium-based structure for API 5L X52 steel in 0.5 M H_2_SO_4_, where halogens were exchanged by a thiocyanate (SCN^–^) group. The authors carried out DFT studies, where the HOMO was located over S and N of the thiocyanate anion and the LUMO was located through the imidazolium ring, which gave the molecule the capacity to donate and accept electrons from other chemical species. From the EDS analysis, the orthorhombic section of the Euler space that conforms to the steel material was calculated, which suggested that the IL presented the preference to interact with specific steel planes and emphasized the importance of the texture of the steel crystallographic surface.

In another study on CIs based on ILs with halogens by Sakki et al.,^78^ for API 5L B steel in 0.5 M H_2_SO_4_, three structures featuring 1-(2-oxopropyl)pyridin-1-ium iodide with three functional groups were evaluated: phenyl (**C1**), thiophen-2-yl (**C2**), and ferrocenyl (**C3**) (see Table 4). Preliminarily, the WL tests confirmed that the inhibiting behavior had the following trend: **C3** ≈ **C1** > **C2**. Afterward, the PDP and EIS tests revealed the same behavior pattern. In order to study the stability of the inhibiting film on the API 5L B steel, the authors performed the analysis at different temperatures at maximal C_INH_, finding the same trend in addition to the increase in the *IE* as a function of the temperature.

The theoretical calculations showed that in the three cases, the HOMO was located over the I^–^ anion, being capable of donating electrons to the metal, whereas the LUMO, in the case of **C1** and **C2**, was distributed over the pyridinium ring. In contrast, in the case of **C3**, it was distributed from the pyridinium ring to the ferrocene ring, with these being the molecular parts that accept the electrons from the metal surface. According to the suggested adsorption mechanism, it was proposed that the I^–^ ion worked synergistically with the cation through the formation of a film with an excess of negative charge with a dipole oriented toward the metal positive sites, thus allowing further electrostatic attraction of the cation. In the case of **C3**, the ferrocene organometallic ring could be protonated through Fe and be attracted by previously adsorbed I^–^ ions. This event can also occur through the transfer of electrons in chemisorption processes through the carbonyl group (with pairs of lone electrons in O) and aromatic rings with the possibility of back-donation through the charge transfer from the Fe d-orbitals to the CI empty π orbital.

Also, it has been confirmed that the ILs with quaternary ammonium are effective at inhibiting the corrosion of API steels in H_2_SO_4_. In 2018, Olivares-Xometl et al.^79^ studied four ILs derived from quaternary ammonium with alkyl-sulfate anions as CIs of API 5L X60 steel in 1 M H_2_SO_4_. Preliminarily, WL tests were carried out, finding an increase in the *IE* (∼10%) with the increasing number of carbon atoms of the cation alkyl chain: dodecyl < tetradecyl < hexadecyl. In contrast, the decrease in the anion alkyl chain from ethyl to methyl displayed a nonsignificant reduction of the *IE* (∼2%) at 100 ppm of IL. The authors attributed the *IE* of the evaluated CIs to the electrostatic attraction processes and formation of coordination bonds between the N, O, and S heteroatoms and the steel surface. At the same time, it was suggested that the mechanism of the CIs in H_2_SO_4_ corresponds to a mixed behavior pattern with anodic predominance, i.e., considering a higher contribution of the alkyl-sulfate anions in the steel protecting film. As a continuation of the foregoing research work, Likhanova et al.^80^ studied the influence of adding substituents to quaternary ammonium cations by using alkyl chains with 6 and 8 carbon atoms; furthermore, the anionic behavior between the organic ions adipate and ethyl sulfate to protect API X60 steel in 1 M H_2_SO_4_ was compared, calculating *IE*s of 71 and 83%, severally. The authors found that the *IE* of the IL with ethyl sulfate as the anion and octyl alkyl chain as the cation diminished in 12% with respect to the IL with adipate anion and hexyl cation. From the previous results, it can be deduced that the proximity of the S and O heteroatoms, accompanied by an alkyl chain in the ethyl sulfate anion, transformed the CI into a more competitive molecule to occupy available steel sites in the acid medium; likewise, it was observed that the position of the carboxylic groups in the adipate ion could make difficult their orientation due to their location at the opposite ends of the anion molecule.

In another study on ILs with ammonium cation for inhibiting the corrosion of API 5L X52 steel in 0.5 M H_2_SO_4_^81^ carried out by the same research group, the influence of three different counterions derived from carboxylic acid was compared: laurate, anthranilate, and oleate. Based on the LPR and PDP tests, it was observed that the *IE* depended on the anion, finding the following trend: anthranilate ≈ laurate > oleate, which was attributed to the presence of the aromatic ring with an amine group in the anthranilate anion, which promoted a better orientation of the triethyl-methylammonium cation. Likewise, the authors emphasized the *IE* (74%) of the compounds with anthranilate and laurate anions, even under hydrodynamic conditions (N_RE_ = 4500), which stemmed from the fact that the transport phenomena in the system favored the availability of the inhibitor molecules toward the metal surface.^83^ This behavior pattern was observed by Olivares-Xometl et al.,^54^ demonstrating that an IL with acetate anion is more efficient that an IL with laurate at N_RE_ = 0, protecting the metal surface with *IE* of 68% due to the fact that its smaller size allowed better orientation of its heteroatoms toward the API 5L X52 steel in 1 M H_2_SO_4_. From the previous results, it was concluded that the combination of ILs with quaternary ammonium cations and short alkyl chains as substituents displayed enhanced inhibition behavior without flow when the anions are derived from short chain carboxylic acids, whereas with the presence of anions with long chains or aromatic rings, the inhibition behavior was improved under hydrodynamic conditions.

Recently, much interest has been set in sulfonium-based ILs due to properties such as relatively low viscosities, high conductivities, and wide electrochemical window.^90,91^ Díaz-Jiménez et al.^83^ studied the corrosion inhibition process of API 5L X52 steel in 1 M H_2_SO_4_ in the presence of a sulfonium IL. The authors reported that the combination of the decyldimethyl sulfonium cation and iodide anion inhibited the corrosion process and the formed film was resistant to the τ of the system laminar and transitory flow whose behavior was attributed to the I^–^ synergy, which participated as a bridge between the metal positive charge and sulfonium cation. With the N_RE_ increase (from 4000 to 5000), there was an important decrease of *IE* ≈ 20% due to the CI desorption, which allowed the diffusion and later adsorption of the aggressive ions in the medium on available active sites.

In the past few years, the use of polymers as anticorrosive materials has attracted special attention, because they have more than one functional group in the same molecule. In this context, polymeric ILs or poly(ionic liquids) (PILs) have stood out because of their additional advantage before conventional polymeric compounds by possessing ions with a polymeric structure. Gómez-Sánchez et al.^84,85^ proposed the study of three PILs as CIs for API X60 steel in 1 M H_2_SO_4_: PILs with cations derived from acrylamide, vinylpyrrolidone, and 1-alkyl-3-vinylimidazolium, and bromide and imidazolate anions (Table 4). According to the electrochemical evaluation, the best *IE* was displayed by PILs with different N-heteroatom and/or N-heterocycle blocks, thus confirming the synergistic effect of the bromide ion as well as the inhibiting activity of the substituted imidazolium ring. However, with the temperature increase, the PIL 1-butyl-3-vinylimidazolium with imidazolate presented slower adsorption–desorption rate from the metal surface than those of the other PILs. The authors suggested that their polymeric nature gives the possibility of producing CIs with different conformations (e.g., linear and/or crisscross), thus enabling the improvement of the CI orientation and adsorption process as well as the stability of the protecting film.

An option of CIs based on nontoxic-inorganic-anticorrosive pigments was studied by Chaudhry et al.^86,87^ who employed nickel ferrite (NiFe_2_O_4_) [**NF**] and nickel zinc ferrite (Ni_0.5_Zn_0.5_Fe_2_O_4_) [**NZF**] nanoparticles to inhibit the corrosion of API 5L X80 steel. The authors suggested that the pigments displayed a cathodic behavior pattern, which was attributed to the activity of the Ni^2+^, Zn^2+^, and Fe^2+^ species that can protect the metal by generating galvanic cells on the surface. For both pigments, the authors observed that the *IE* presented inverse behavior to the C_INH_, where their inhibiting effect was attributed to the adsorption or electrodeposition reaction of cations on the steel surface; however, the increasing C_INH_ favored the formation of galvanic cells on the anodic sites, which increased the Fe dissolution. In both studies, the adsorption of ferrite, zinc, and nickel on the steel surface was confirmed. The results indicated that at low C_INH_, *IE* > 70% was obtained. Notwithstanding, the environmental impact of using these pigments is unknown.

The necessity of replacing more and more conventional organic CIs with “green” CIs has prompted the modification of chemical structures according to the current environmental standards. Likewise, it is important that today’s research works also consider environmental interest variables such as toxicity levels, bioaccumulation, biodegradability, etc. in order to draw a more realistic panorama of functional groups that can be both efficient and safe to be used as CIs.

#### 3.1.3 Corrosion Inhibition of Sulfamic Acid Environment

Sulfamic acid (H_3_NSO_3_H), also known as amino sulfuric acid, is an intermediate product between sulfuric acid (H_2_SO_4_) and sulfamide (H_4_N_2_SO_2_). This acid is employed in diverse applications such as heterogeneous catalyst to carry out organic synthesis, esterification, and sulfation reactions as well as precursor of other chemical compounds of industrial importance.^92,93^ In the drilling process to extract oil and gas, it is usually used to reduce the pH of the employed fluids and extraction compounds before their later elimination because of main benefits such as easy handling, solubility, and low corrosivity.^94,95^

H_3_NSO_3_H solutions (5–10%) are also applied as alternative electrolytes and eco-friendly substitutes of HCl to remove scales and deposits of metal oxides from the internal surface of equipment and steel pipeline systems to prevent the metal walls from reacting with the transported fluid, thus promoting undesirable secondary reactions. Although its attack is less aggressive than that of other acid media such as HCl or H_2_SO_4_, it also represents a corrosive medium for metal structures.^92−95^ In the past decade, only two CIs based on pyridopyrimidine^94^ derivatives and a mixture of polymers/Au nanoparticles^96^ have been developed to reduce the corrosion of API steels in H_3_NSO_3_H at 5%.

Abdallah et al.^94^ published an interesting study, where two new structures derived from pyridopyrimidine were synthesized (see Table 5), evaluating their behavior as CIs of API 5L X52 steel. The authors employed three electrochemical techniques (PDP, EIS, and EFM) to validate the obtained results, finding that both compounds presented *IE*_max_ values of 92.2 and 86.4% at 298.15 K. Furthermore, the metal-inhibitor system was evaluated at 323 K by PDP and a slight diminution of *IE*_max_ (∼5%) was observed, which was associated with higher surface activity and desorption of the CIs. Unlike other studies, both CIs presented a slightly significant *IE* difference despite the presence of a hexadecyl (−C_16_H_33_) group, which could have been attributed to the number of functional groups that take part in the adsorption processes. According to these authors, the compounds presented multiple adsorption centers through the benzyl ring (with π electrons) and S, N, and O heteroatoms. The DFT calculations of compounds **I** and **II** showed the distribution of the MOs throughout the whole molecule, except in the 4-methoxyphenyl group. In general, the MOs are located in regions, where there are heteroatoms and aromatic rings, however, both behavior patterns are attributed to the employed base and functional (BOP/DNP). Afterward, the MD simulation suggested the adsorption process of both molecules, where **I** had planar adsorption with the 4-methoxyphenyl leaving group. In contrast, **II** displayed totally planar adsorption parallel to the Fe (110) surface, thus supporting the slight increase in the *IE* in comparison with that of **I**, which was obtained by electrochemical tests. As for Yassin et al.,^96^ these researchers analyzed the potential of incorporating gold nanoparticles (**Au**-**NPs**) to enhance the inhibiting behavior of a mixture consisting of poly(vinyl chloride-*co*-vinyl acetate-*co*-2-hydroxypropyl acrylate) (**PVVH**) and poly(ethyl methacrylate) (**PEMA**) for API 5L X70 steel. From the electrochemical tests at 298 K, it was observed that the material hybridization did not increase the *IE* as expected, showing maximal increments of 5% with respect to the polymer mixture; in contrast, at 328 K, the increments were of ∼9%.

**Table 5:**
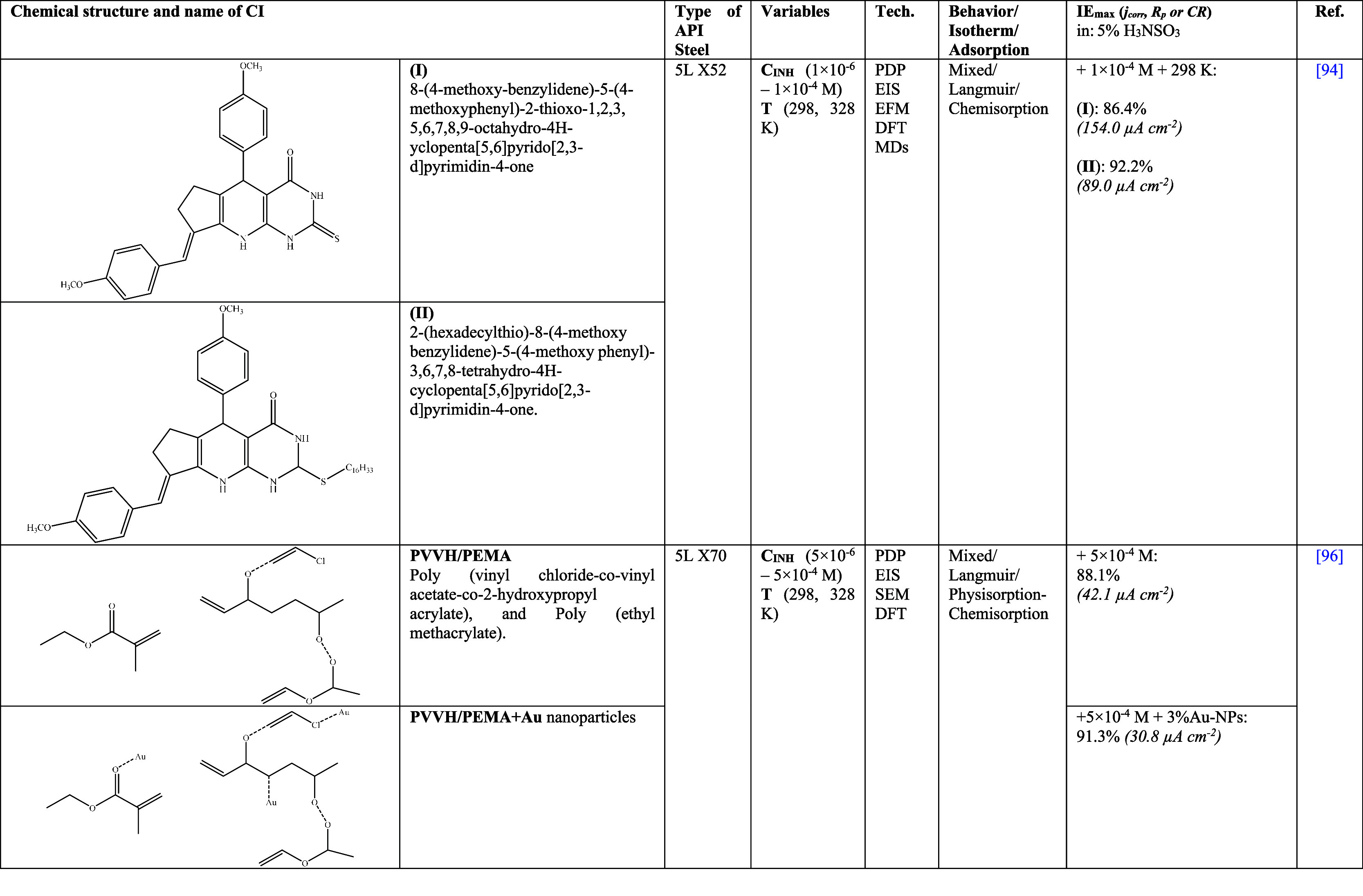
Some Examples of Corrosion Inhibitors for API Steels in Sulfamic Acid (H_3_NSO_3_)^94,96^

In the DFT calculations, a twisted conformation between the different PVVH blocks was observed, where the Au atoms were linked only with the O atoms without considering the CI. However, the MEP analysis suggested that the Au atoms presented negative charge, which was a behavior pattern that resulted in a behavior pattern that was opposite to the one revealed by the Mulliken charges and the very nature of the metals. On the other hand, the HOMO was distributed over C=C–OH and C=C–O–Au, whereas the LUMO was located over O atoms of **PVVH/PEMA** and Au atoms of **PVVUH/PEMA+Au-NPs**. The authors associated the inhibiting behavior with the protonation possibility of the **PVVH/PEMA** heteroatoms and their attraction toward the metal surface through unpaired and π electrons. Even when the corrosion degree caused by sulfamic acid (*K*_a_ ≈ 1 × 10^–1^) is lower than those of HCl and H_2_SO_4_, the study of compounds capable of mitigating the corrosion effect on API steels is also required. As can be seen in this section, the studied inhibitors are relatively just a few; notwithstanding, it has been found that compounds such as pyrimidinone or acrylates have reached *IE*s above 80%.

### 3.2 Corrosion Inhibition of Sweet Environment

Sweet corrosion occurs when steel is deteriorated by an environment consisting of carbon dioxide (CO_2_) and is affected by various factors such as the basicity increase, temperature, medium characteristics, and metal type as well as by the flow dynamics, being considerably more corrosive in the presence of brine.^97^

In gaseous state, CO_2_ is not corrosive itself; however, in an electrochemical process that implies corrosion, CO_2_ gas has to be dissolved in aqueous phase and a hydration reaction has to take place, where a more reactive chemical species is formed: carbonic acid (H_2_CO_3_); afterward, dissociation reactions occur to form bicarbonate (HCO_3_^–^), carbonate (CO_3_^2–^), and hydrogen (H^+^) ions.

The foregoing reactions promote acid pH in the fluid, which in contact with steel results in the formation of corrosion products such as ferrous carbonate (FeCO_3_) and the release of hydrogen gas (H_2_).^98^ The general process of the sweet corrosion of steel is described by Reaction 5:^99^
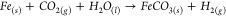
5

The proposals of CIs for sweet corrosive environments have been diverse, finding compounds from hydrolyzable tannins,^100^ gemini nonionic surfactants synthesized from avocado oil,^101^ drugs,^102^ carbohydrates with synergistic agent^103^ to synthetic chemical compounds,^104,105^ ionic liquids (ILs),^106^ organically functionalized graphene oxide,^107^ and a hybrid polymer–metal composite.^108^Table 6 presents the results obtained for CIs of the corrosion of API steels in the presence of 3.5% CO_2_ and NaCl at 3.5%.

**Table 6:**
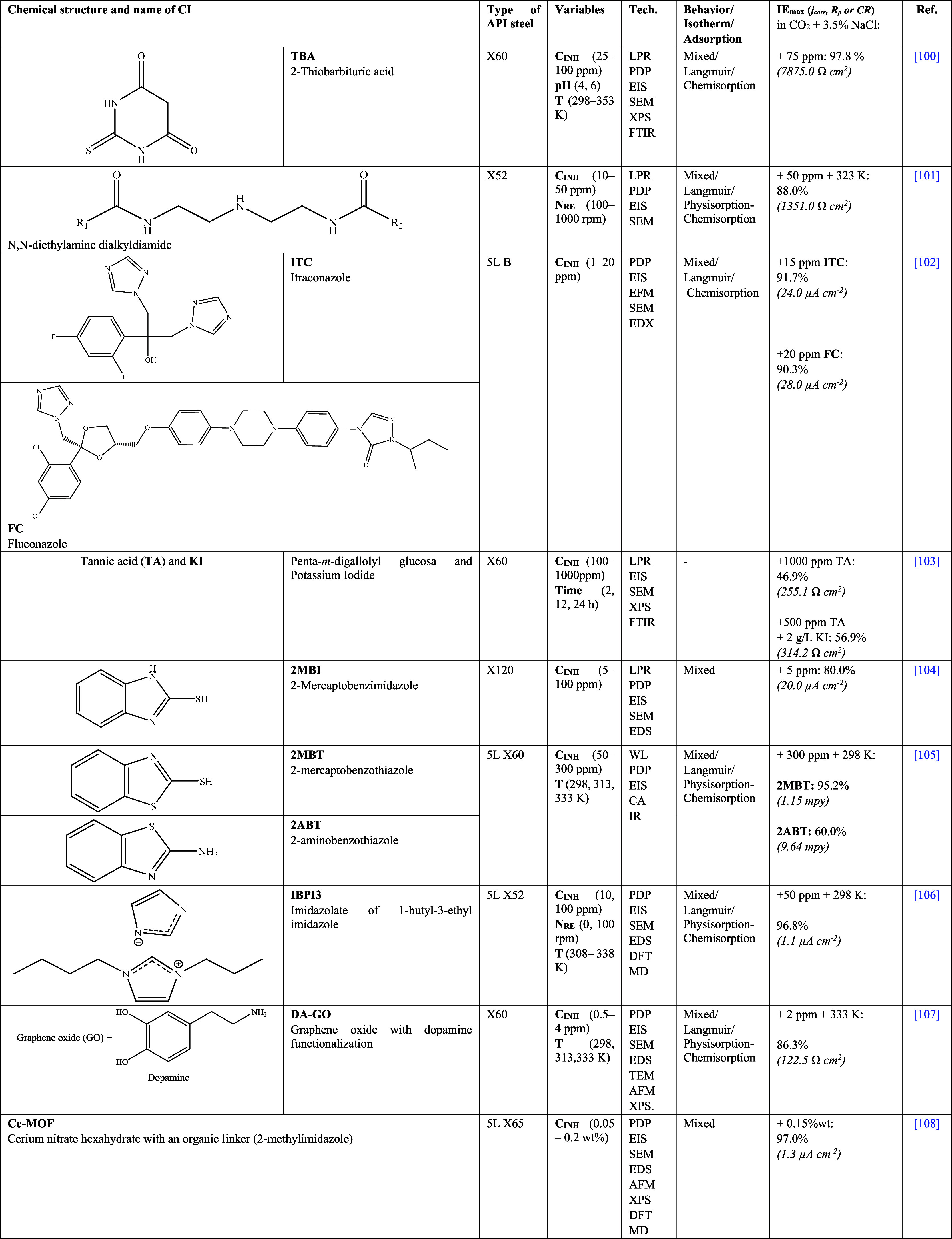
Various Corrosion Inhibitors for API Steels in a Sweet Environment (CO_2_ and NaCl)^100−108^

The first reported studies were carried out by Usman et al.^100,103^ who evaluated the inhibition behavior of 2-thiobarbituric acid (**TBA**) and tannic acid (**TA**) for API X60 steel; despite the obtained *IE*s, the literature indicates that these chemical compounds are considered as toxic,^109^ which makes their application as CIs unlikely. In contrast, Cruz-Zabalegui et al.^101^ synthesized a CI for API X52 steel derived from a mixture of oleic, palmitic, and linoleic acids, starting from avocado residues, as a more environmentally friendly option. In the CI tests, the dependence of the *IE* on the **TBA** C_INH_ was observed; in contrast, **TA** did not present a defined *IE* behavior pattern as a function of C_INH_. Additionally, potassium iodide (**KI**) was added in order to observe its synergistic effect, which diminished when the level of **TA** C_INH_ was increased. Afterward, the film stability was analyzed up to 353 K and immersion times above 72 h, where the **TBA***IE* displayed negligible changes (∼2%). In contrast, the *IE* in the **TA** + **KI** presence showed a constant behavior pattern at 24 h (∼90%), i.e., a stable inhibiting film was achieved. As for Cruz-Zabalegui et al., these authors analyzed the flow rate effect at 333 K on C_INH_, finding that even with the presence of hydrophilic and hydrophobic groups, the diminution of *IE*_max_ occurred as a function of the increase in the angular velocity of the rotating disc. This behavior was provoked by τ with the increase in the mass transfer (aggressive ions and CIs) and desorption of the inhibiting film. The foregoing was supported by SEM, where, under static conditions, the formation of a more compact film with fewer pores was observed; in contrast, at 500 rpm, an irregular surface with CI uncovered regions was identified. In this context, Fouda et al.^102^ evaluated compounds with antifungal properties such as itraconazole (**ITC**) and fluconazole (**FC**) in their commercial forms as CIs of API 5L B steel. However, their use could imply the increase in the resistance to fungicides of determined populations of pathogenic fungi and in the long term, a potential health problem.^110^ The authors reported that C_INH_ of 15 and 20 ppm is required to obtain *IE*s of 91.7 and 90.3%, respectively. Afterward, the inhibition process was analyzed by increasing the temperature from 298 to 328 K, where a drastic *IE* diminution of 73% occurred. It was suggested that the protection of the metal surface was improved due to the formation of iron nitrides (Fe_2_N and Fe_8_N) coming from the amount of N atoms present in the molecule and their interaction with Fe. In the proposed inhibition mechanism, the CIs interacted with API steel through already adsorbed and hydrated aggressive ions, which provided the surface with an excess of negative charge. In contrast, structures based on heterocycles such as 2-mercaptobenzothiazole (**2MBT**), 2-aminobenzothiazole (**2ABT**), and 2-mercaptobenzimidazole (**2MBI**) (see Table 6) were studied along with different API steels by Larios-Galvez et al.^104^ and Ahmad-Zamani et al.^105^ According to the data reported in both studies, the authors agreed that these CIs presented a decrease in the *IE* as a function of C_INH_ due to steric effects between the rings adsorbed on the surface and those present in the solution core. In the case of Larios-Galvez et al., these authors evaluated API X120 steel by means of thermomechanical treatments different from enrolling, cooling, and reheating, reporting that API X120 steel displayed a decrease in the *IE* at higher **2MBI** C_INH_; such behavior was related to the different compositions of martensite, austenite, bainite, and ferrite present in the microstructure of the treated steels. Based on the foregoing, the adsorption processes were associated with the functional groups of each molecule such as −SH, −NH_2_, and S and N heteroatoms and aromatic rings. In the case of Ahmad-Zamani et al., these researchers confirmed the increase in the hydrophobicity of the API 5L X60 steel surface in the presence of **2MBT** by means of the Contact Angle technique and, at the same time, the formation of micelles from the Volta potential obtained by mapping via the Scanning Kelvin Probe. In addition, the authors suggested that inhibitor **2ABT** allowed the adsorption of CO_2_ in its structure through the −NH_2_ group.

As for Ontiveros-Rosales et al.,^106^ these authors analyzed the imidazolate of 1-butyl-3-ethyl imidazole (**IBPI3**) as potential CI for API 5L X52 steel in NaCl saturated with CO_2_. According to electrochemical techniques, the *IE* showed a dependence on C_INH_ up to 50 ppm. On the other hand, the hydrodynamic conditions at C_INH_ of 100 ppm revealed irregular behavior, which could be attributed to the instability of the inhibiting film. Afterward, the temperature analysis showed the diminution of the *IE* up to ∼43% with 50 and 100 ppm, which stemmed from the desorption of the inhibiting molecules. These results were supported with DFT theoretical calculations, considering the interaction between the optimized IL molecule and a cluster of 6 Fe atoms. From the geometrical optimization, a bond energy value of 3.549 kcal mol^–1^ was obtained, and the authors indicated that the dissociation of ionic species allowed their interactions with Fe separately; it is worth emphasizing that the HOMO and LUMO lobes were located over the N atom, with less contribution over adjacent C atoms. In contrast, in the formation of the Fe–N bond, the redistribution of the lobes over the cluster took place from available electrons in the heteroatoms present in **IBPI3**, as suggested by the MEP analysis. The DFT calculations were extended employing MD, which confirmed the parallel adsorption of the imidazolate ring through a single N atom for Fe in different conformations such as 100, 110, and 111. In their proposed adsorption mechanism, the authors suggested that the adsorption process occurred through charge transfer processes from **IBPI3**, mainly through the imidazolate ion by means of physicochemical interactions with the possibility of forming Fe–N covalent bonds.

In the past decade, research works employing two-dimensional graphene oxide (**GO**) sheets have thrived in different applications due to characteristics such as high mechanical and thermal resistance, electrical conductivity, high impermeability, low cost and high surface.^111^ In this context, Haruna et al.^107^ studied the functionalization of GO with dopamine (**GO-DA**) as potential CI for API X60 steel in saturated CO_2_ in a NaCl solution at 3.5%. Through electrochemical tests, it was observed that the optimal inhibition concentration was 2 ppm, which was associated with higher inhibition activity by occupying more available active sites of the metal surface through interactions between adsorbed species and **GO-DA** molecules at the metal interface. Afterward, the analysis at 2 ppm and higher temperatures showed a slight increase in the *IE* (∼5%), which was related to the thermal stability displayed by **GO-DA** and the increasing formation of Fe_2_CO_3_ at high temperatures. In contrast, under hydrodynamic conditions at different temperatures, an *IE* increase of up to 96% was observed, which was attributed to a higher diffusion of inhibiting molecules toward the steel surface, which favored the **GO-DA** adsorption.

The surface morphology identified by SEM analysis in the **GO-DA** presence did not display a sheet arrangement, which was probably due to the adsorption, structural reorganization, and interaction with the sweet medium. This fact was also supported by the XPS technique, where characteristic chlorine and iron peaks were observed and confirmed the formation of inorganic chlorides such as FeCl_2_ and FeCl_3_ as well as the absence of organic chlorides. Additionally, the C–N^+^ signals suggested the formation and adsorption of the protonated form of **GO-DA**, which interacted with Cl ions that were previously adsorbed on the metal surface by physisorption and chemisorption, where the **GO-DA** heteroatoms shared pairs of lone electrons with the empty Fe d-orbital; likewise, backdonation processes were considered.

Another proposal of composite materials was supported by Anadebe et al.^108^ who reported the synergistic effect of cerium nitrate hexahydrate with an organic connector based on 2-methyl-imidazole (**M-IMI**) as nanohybrid corrosion inhibitor (**Ce-MOF**) for API 5L X65 steel. According to the electrochemical results, an *IE* increase of the organic connector (**M-IMI**) with respect to the C_INH_ of **Ce-MOF** of up to ∼70% was observed, which was related to the joint action of ions/metal groups and **M-IMI** to form an organic/inorganic film on the steel surface. Notwithstanding, a slight *IE* decrease (∼7%) at 2.0 wt % of **Ce-MOF** occurred, which was ascribed to the clustering of **Ce-MOF** molecules that reduced the availability of the molecule active sites and/or the CI desorption. Afterward, by means of a field emission scanning electron microscope, the formation of cracks and deep pitting was observed, which was associated with the dissolution of the ferrite phase of API 5L X65 steel in the blank case. In contrast, in the **Ce-MOF** presence, a dense and almost intact surface was revealed, which reduced the attack of the medium aggressive ions. Additionally, micrographs of the cross section were obtained, where a corroded edge with scales and iron oxides deposited after exposure to the sweet corrosive medium was identified. On the other hand, in the **Ce-MOF** presence, a stable film on steel was obtained; likewise, these micrographs also allowed the measurement of the film thickness in both cases, observing that the thickness of the **Ce-MOF** film tended to double with respect to the blank thickness, which was associated with the CI accumulation. The authors carried out a computational analysis by DFT, suggesting that the interaction and anchoring regions of the molecule were located in the Ce atoms and imidazole rings, specifically in the N and double bonds. However, it was inferred that the imidazole rings could be rearranged to favor the planar adsorption with respect to the metal surface as suggested by the MD analyses; this behavior pattern was also attributed to the addition of a more electropositive metal to the **M-IMI** organic matrix. In this work, the use of an adaptive neuro-fuzzy inference system (ANFIS) was specially emphasized, which allowed the integration of automated learning methodologies to predict efficiencies. The model was fed with 80% of the experimental data obtained in the same study and the remaining 20% was calculated by means of the ANFIS predictive model, finding that with this system, reliable results can be obtained for the analysis of CIs. As observed, many of the compounds employed in sweet medium turned out to be good proposals by not requiring high concentrations (<100 ppm) in addition to have common molecules based on aromatic rings such as pyrimidine, phenyl, piperazine, triazole and imidazole derivatives (benzimidazole and imidazolium/imidazolate) with at least two heteroatoms (N, O, and/or S) that provided reactive sites to the CI. These results have allowed the proposal of new CI structures that could be equal or better in the inhibition performance at lower concentrations for this corrosive medium. Unfortunately, due to the fact that some of the analyzed structures are toxic and others have not been studied about their environmental effects, their future applicability is not feasible.

### 3.3 Corrosion Inhibition of Sour Environment

In addition to CO_2_, oil transport fluids also contain a significant amount of H_2_S, where both chemical compounds are considered as the main origin of internal corrosion, which along with different variables and process parameters can prompt different scenarios; for example, the delay of the corrosion rate can be favored by the presence of corrosion products located on the metal surface, generated by a temperature increase, partial pressure values and pH; in contrast, such effect can be altered by the increase in the flow rate and presence of organic acids.^112^

The corrosion process in a H_2_S medium is commonly known as sour or bitter corrosion, where this medium favors highly acid media. The formation mechanism of hydrogen sulfide (H_2_S) occurs during the hydrocarbon extraction, by sulfate reducing bacteria, or during the thermal cracking of sulfur-containing compounds. Its interaction with metal alloys provokes different types of damage that ends up with the metal loss.^113−115^ The corrosion mechanism by H_2_S forms a multilayer film of corrosion products consisting of a hydroxide external layer, an intermediate sulfide layer, and an internal oxide layer. As for the corrosion rate, it is directly related to the production of iron sulfide (FeS) (Reactions 6–9), which is a nonstoichiometric corrosion product^116^ that can form different compound types such as mackinawite (Reaction 9), cubic FeS, amorphous FeS, and troilite, whose formations depend on the H_2_S concentration, pH, and temperature:^114,115^
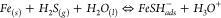
6
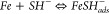
7
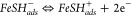
8
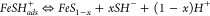
9

In systems where CO_2_–H_2_S coexists even with H_2_S at low amounts, CO_2_ leads considerably the corrosive behavior pattern, generating an environment that is more complicated to be controlled due to the fact that their partial pressures exert a prevailing effect on the steel anodic dissolution and promote pitting corrosion on the metal surface.^117^ Especially, this medium is one of the most complicated ones to be controlled regarding API steels. In this context, it could be said that there are relatively few studies on CIs for API steels in a sour corrosive medium. From the evaluation of some CIs, it was found that their inhibition process requires high concentrations to reach *IE* ≥ 80%.

In H_2_S medium, nitrogenated compounds have been employed as CIs for API steels as shown in Table 6. In order to contribute to the understanding of this medium, Sabzi et al.^115^ analyzed a synthetic CI consisting of 2-mercaptobenzothiazole for API X60 steel at 10–20 ppm of H_2_S. The authors observed that by diminishing the concentration of 2-mercaptobenzothiazole, the E_OCP_ was displaced toward more negative values, i.e., the susceptibility of API X60 steel grew in the presence of an increasing amount of H_2_S corrosive ions. This result suggested that C_INH_ changed the structure of the electrical double layer. The PDP electrochemical tests supported this behavior, which revealed that the anodic and cathodic reactions taking place on API X60 steel diminished as a consequence of the reduction of the current density of the metal surface due to the presence of the CI. The adsorption of 2-mercaptobenzothiazole increased the charge transfer resistance toward the metal surface because of the formation of a protecting CI film with an *IE*_max_ of 97% at 10 g L^–1^. However, the SEM–EDS analysis in CI presence evidenced localized corrosion on the API X60 steel passive film. Likewise, Díaz et al.^118^ analyzed carboxyethylimidazoline as CI for API X120 steel in H_2_S (Table 7). The authors reported the reduction of the polarization resistance in the presence of carboxyethylimidazoline with an *IE*_max_ of 97% (160 μmol L^–1^). Nevertheless, the inhibiting film presented desorption processes at a C_INH_ of 332 μmol L^–1^; such behavior was related to the length of the alkyl chain consisting of 14 carbon atoms. Additionally, by EIS, the authors supported the formation of a film consisting of iron sulfide as corrosion product, where the reduction of the semicircle diameter of the Nyquist spectra was associated with the possible growth of the iron sulfide film up to critical thickness with its further cracking and detaching from the metal surface. An alternative option of a friendlier CI was offered by Hamri et al.^114^ for API 5L B steel in 15 ppm of H_2_S. The authors employed the extract of the medicinal plant Henna (*Lawsonia inermis L. o Lawsonia Alba*) that is rich in phytoconstituents of different types with four main chemical compounds: 2-hydroxy-1,4-naphthoquinone (lawsone), 3,4,5-trihydroxybenzoic acid (gallic acid), methyl gallate (tannic acid), and α-d-glucose. The electrochemical techniques revealed efficiencies from 75 up to 92% at 0.05 g/L and ≥2.5 g/L of extract, respectively. The inhibition behavior displayed by the Henna extract was related to a softened and dense physical barrier between the corrosive medium and the metal, which was formed by physisorption mechanisms. The DFT calculations indicated that the distribution of the MOs took place through the aromatic rings, related to the presence of π electrons from the conjugated bonds.

**Table 7:**
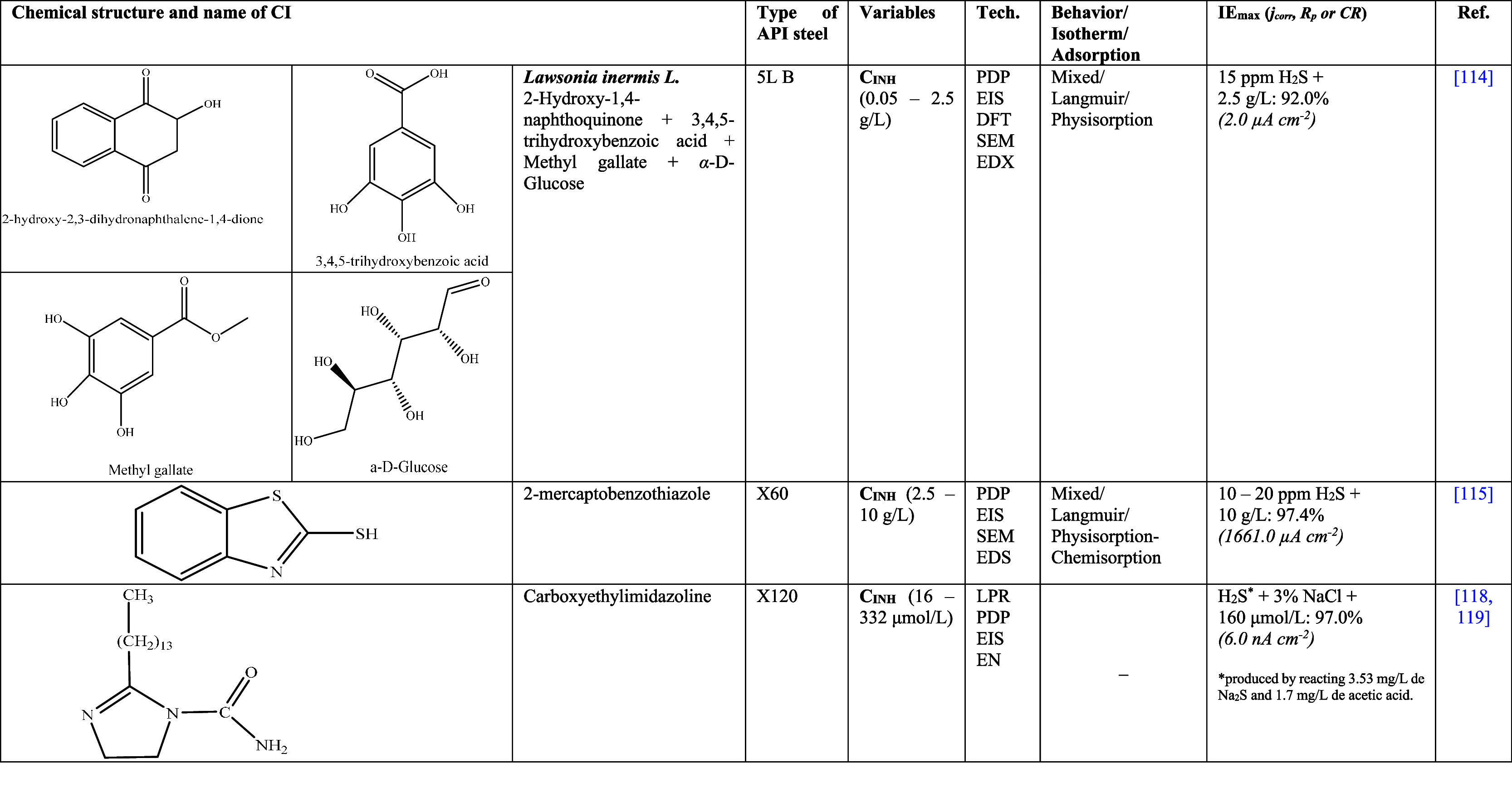
Some Corrosion Inhibitors for API Steels in Hydrogen Sulfide (H_2_S)^114,115,118,119^

As can be seen, research works on CIs for API steels in this medium are limited despite the fact that H_2_S represents a constant problem for the oil industry at any exploration and oil and gas production stage. Notwithstanding, the structures of the studied CIs were based mainly on aromatic rings that could be linked to heterocycles (N and S) and/or hydroxyl, carboxyl, or amide functional groups, just to mention a few, reaching *IE* > 90% at relatively high C_INH_, thus offering an opportunity area for the research and development of new inhibitors capable of mitigating this problematic situation.

### 3.4 Corrosion Inhibition of Saline Environment

In the oil and gas industry, production water represents the largest volume waste stream in oil and gas production operations in most offshore platforms.^120^ Production water, as a residual subproduct of formation and injected water, generates a complex mixture of inorganic ions (sodium, chloride, calcium, magnesium, potassium, bromide, bicarbonate, sulfur, and iodide), metals, radioisotopes, a wide variety of hydrocarbons, and a mixture of low-molecular-weight carboxylic acids. The physicochemical properties of production water vary depending on the reservoir type, geological age, depth, geochemistry of hydrocarbon formation, chemical composition of the oil and gas phases in the reservoir, and/or of the chemical products that are added to enhance production. The chemical composition and pH of water production are fundamental factors in their corrosive effect. In addition, the temperature and pressure changes can generate the formation of difficult-to-control scales due to the high content of sulfate and carbonate ions as well as the high concentrations of calcium ions in production water, which provokes the precipitation of calcium carbonate (calcite) and calcium sulfate (anhydrite). The corrosion control in this type of alkaline medium requires the use of high CI amounts.^120^

In the literature, different alkaline solutions have been reported such as synthetic brines (NACE ID196/1D182), NaCl, or acetic acid solutions (NACE TM0177–86).^121^ Unlike sweet corrosion, the proposals of “green” CIs for saline media have been limited to a couple of natural compounds,^121,122^ synthetic compounds,^123−126^ and ILs with cations such as pyridinium and quinolinium,^127,128^ thiazinium,^129^ and ammonium,^130^ among others. Also, the proposal of an inorganic CI based on Ni was found.^86^Table 8 shows CIs studied in the corrosion inhibition of API steels exposed to saline media. In 2021, Espinoza-Vázquez et al.^121^ studied *preussomerin G*, a spirodioxinaphthalene obtained from *Pyrenochaetopsis sp*. T1–41 (Plesosporales), as a CI obtained from a fungal source for API 5L X70 steel in 3–5% NaCl with and without 0.5% acetic acid (Table 8). From the results of the WL, PDP and EIS tests, it was found that the CI presented higher *IE*s in saline medium than in the acetic-saline medium, associated with the higher aggressiveness of the first compound ions in the medium. From the foregoing, the authors evaluated the flow effect on the saline medium and found two behavior patterns. In the first one, at low C_INH_ (5, 10, and 20 ppm), an increase in the *IE* up to 15% was observed. In contrast, at higher C_INH_, no significant *IE* change was observed, which was related to the saturation of the metal surface, which limited the adsorption of a higher amount of CI.

**Table 8:**
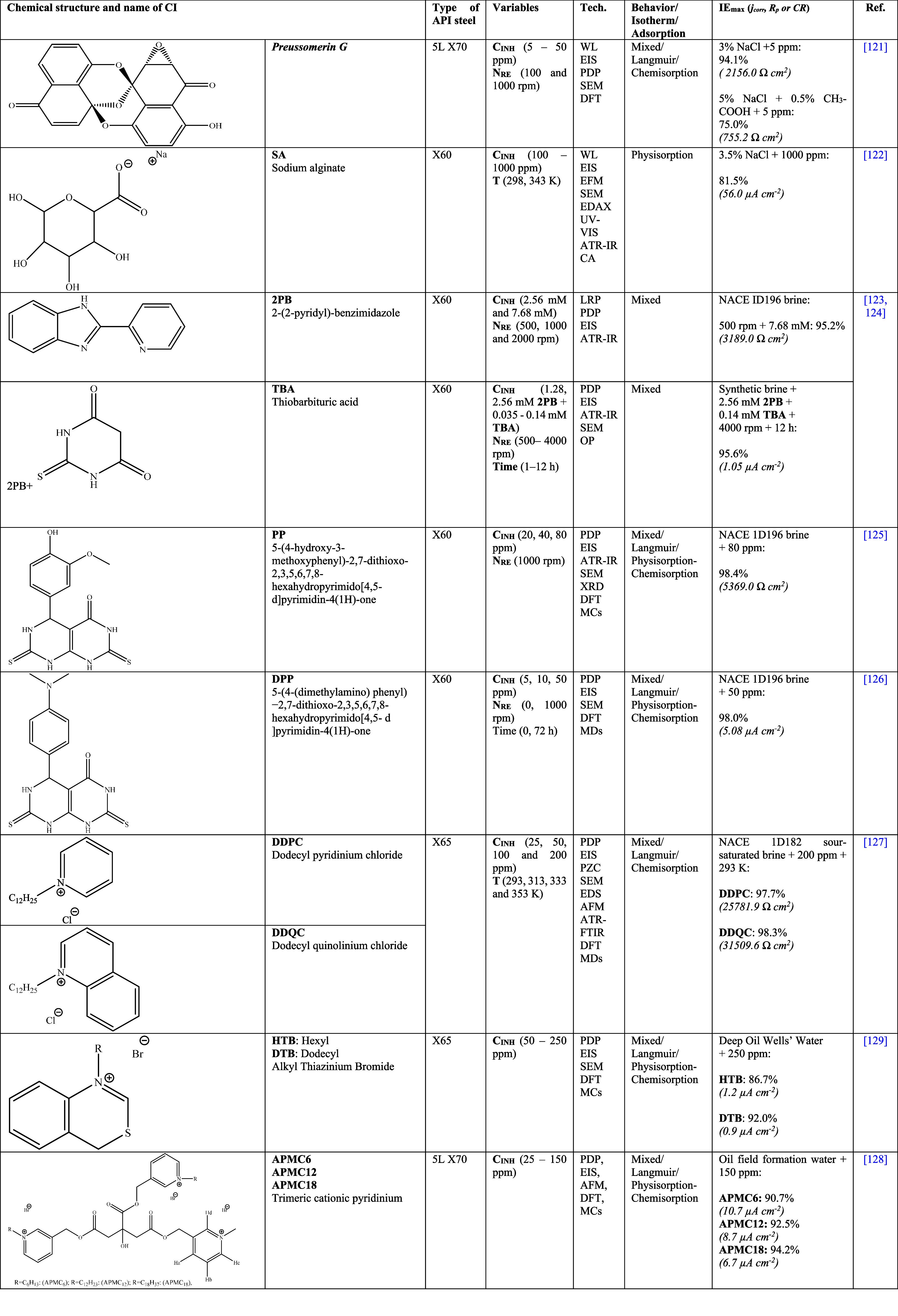

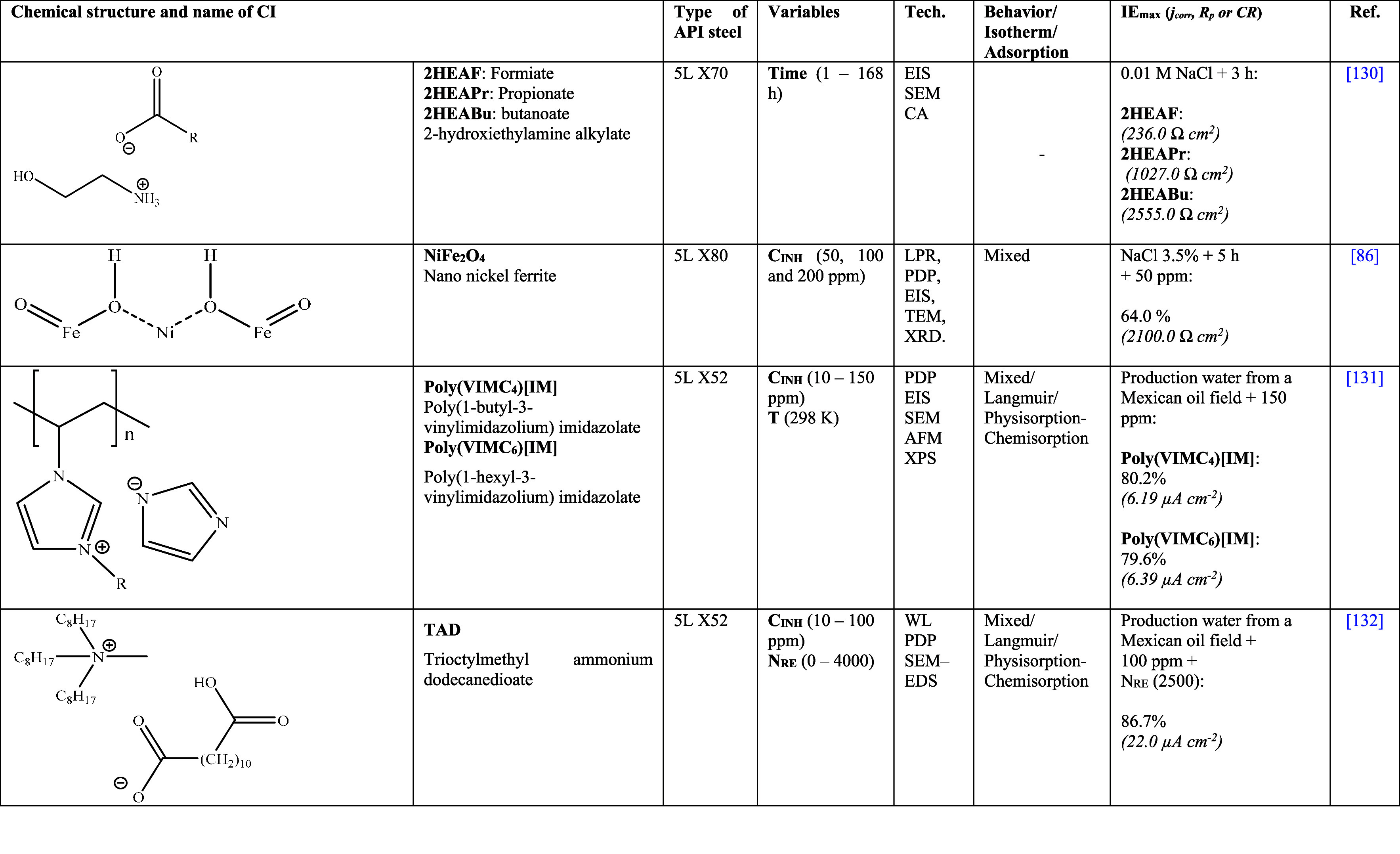
Several Corrosion Inhibitors for API Steels in Saline Media and Produced Water^86,121−132^

Furthermore, it was observed that a desorption process of the CI molecules occurred after ∼40 h, whereas the *IE* was stable up to ∼300 h. The SEM surface analysis of X70 steel in the absence and presence of 50 ppm of CI evidenced a reduction in the surface damage. By means of DFT calculations, the bond lengths obtained from the adsorption of the CI on an Fe_6_ cluster (carbonyl group adjacent to the hydroxyl and ether groups, and an ether group) were analyzed, suggesting that the carbonyl and ether groups presented an excess of electrons, whereas the rings displayed alternated negative and positive charges attributed to their resonance, which facilitated the formation of coordinate bonds between the metal and CI. Another proposal of CI was obtained from natural sources by Obot et al.,^122^ who studied sodium alginate (**SA**) for the corrosion inhibition of API X60 steel in 3.5% NaCl. **SA** is a biopolymer and polyelectrolyte extracted from alga cell walls whose properties such as biocompatibility, biodegradability, nontoxicity, and high solubility result attractive in a CI. The WL analysis showed the increase in the *IE* as a function of the C_INH_, which was associated with the synergy between the Cl^–^ ions and polysaccharide; in contrast, the increase in T favored the CI desorption process from the API X60 steel surface, diminishing the *IE* from 87.23% up to 67.25% at 293 and 343 K, severally. The authors also carried out the analysis of API X60 steel in the absence and presence of CI by attenuated total reflectance infrared spectroscopy, indicating the presence of peaks corresponding to C–O, −OH, and carboxylate groups. Through UV–vis, adsorption peaks at 275 and 340 nm were attributed to the π–π* transition of the **SA** molecules. Thanks to the ATR IR and UV–vis results, it was concluded that **SA** presented physical-type adsorption. In the DFT calculations, the location of the MOs suggested that the **SA** carboxylate groups worked as donors/acceptors of electrons, i.e., these were the most reactive molecule sites, where the interaction with the steel surface took place. In addition, the authors performed an MD simulation, where the **SA** adsorption parallel to the surface was observed by means of carboxylate and H groups from the polymeric skeleton. As for Onyeachu et al., in 2019 and 2020, these researchers ran four different studies on the inhibition of the corrosion of API X60 steel in NACE ID196 brine. To this end, 2-(2-pyridyl)-benzimidazole (**2PB**) was studied by PDP and EIS tests,^123,124^ observing that the *IE* increased with the C_INH_, which was related to the “sealing” of the inhibiting film pores adsorbed on the metal. Under flow conditions, **2PB** displayed stability before the hydrodynamic removal effect, keeping *IE*s at 94% even at 2000 rpm, which was ascribed to the mass transfer process promoted by the adsorption of **2PB**. Afterward, the authors continued with the study of **2PB** and analyzed its synergistic effect with **TBA**, concluding that the H_2_ evolution reaction prevailed with respect to the precipitation of FeCO_3_ in the medium without CI at 500 and 1000 rpm. Additionally, the latter was removed by τ and the CI anodic interaction.

In contrast, at 2000 rpm, a significant increase in the availability of HCO_3_^–^ ions, their dissociation into CO_3_^2–^ and FeCO_3_ nucleation/precipitation processes were considered, which diminished importantly the transfer of electrons to the medium and limited the H_2_ evolution. Notwithstanding, at 4000 rpm, the destabilization of the precipitation process took place due to higher τ and competitive CI attack. On the other hand, the addition of **2PB** favored the mass transport assisted by time, achieving an *IE* up to 84.4%. Individually, the *IE*s of **2PB** and **TBA**, obtained at maximal C_INH_, were equal to 84.4 and 92.0%, respectively. In contrast, with 2.56 mM **2PB** in synergy with 0.14 mM **TBA** at 4000 rpm and 12 h of immersion, only 95.6% was obtained, which was associated with the fast saturation of the metal surface due to the adsorption of multiple CI heteroatoms. The authors suggested that **2PB** and **TBA** were protonated and attracted toward CI ions that were preadsorbed on the metal surface, thus favoring the formation of the inhibiting film. In the SEM micrographs, it was observed that the synergy-exposed surface was denser and with less irregularities than the others. As an alternative to AFM, the authors analyzed 3D optical images, confirming the results obtained by SEM.

In other studies by Onyeachu et al., in 2019 and 2023,^125,126^ the synthesis of two pyrimidines employing **TBA** as precursor was carried out. By comparing the *IE*s of the CIs **PP** and **DPP** at low C_INH_ (5–80 ppm) and 1000 rpm, values ranging from 89 up to ∼98% were calculated. The compound **DPP** was the most efficient (*IE*_max_ of 97%) at a lower concentration than that of **PP**, which was ascribed to the influence of the substituent group dimethylamino present in its chemical structure. From the electrochemical tests, conclusions similar to those of **2PB** and **TBA** were obtained. However, the authors analyzed the stability of the DPP film at 72 h, finding significant diminutions in the *IE* related to the flow rate and competition of aggressive species. Afterward, DPP was analyzed computationally, locating the HOMO and LUMO mainly over benzylimidazole, and by MD, the planar adsorption of benzylimidazole on the iron (110) surface was suggested. Iravani et al.^127^ analyzed the inhibiting effect of ILs based on pyridinium (**DDPC**) and quinolinium (**DDQC**) on the corrosion of API X65 steel in NACE 1D182 brine solution by means of different electrochemical, surface, and computational techniques. From the WL, PDP, and EIS tests, it was observed that the *IE* was stable independently of the C_INH_, temperature, and evaluated time, calculating values from 96%, which were attributed to the strong adsorption of the CI through chemisorption processes.

Furthermore, by potential zero charge, it was determined that the metal surface presented an excess of negative charge; notwithstanding, the most positive value of Antropov’s “rational” corrosion potential (E_r_ = E_OCP_ – E_PZC_) confirmed that the CI adsorption took place on the surface of the API X65 steel. This behavior was supported by the SKP technique, where the distribution of the negative potential due to the CI adsorption was observed. Through 3D images produced by the AFM technique, it was possible to observe important surface damage on the blank as well as the shortening and roughness diminution of the API X65 steel in the presence of CI due to the formation of an inhibiting film. The EDS analyses in the CI absence revealed S and O signals, suggesting the formation of iron sulfides and oxides; also, elemental characteristic signals of the medium (Na and Cl) were found. In contrast, in the presence of CIs, the Fe percentage increased, O diminished, Na and Cl disappeared, and N and C appeared, thus confirming the CI adsorption. Moselhy et al.^129^ studied the inhibition effect of two ionic surfactants based on the cation thiazine with the extension of the alkyl chain (**DHB** and **HTB**) on the corrosion of API X65 steel in deep oil well water. Both CIs showed an increase in *IE* as a function of C_INH_. However, **DHB** presented higher *IE*s than those of **HTB**, which was ascribed to the longer alkyl chain. Afterward, the SEM micrographs revealed the reduction of the surface damage of API X65 steel for a 15-day period of immersion in the CI presence. The authors carried out the analysis of both structures through DFT, suggesting that the HOMO and LUMO were located over Br and the thiazine ring, respectively. By means of MCs, the adsorption parallel to the thiazine ring on the Fe (110) surface was confirmed. The proposal of an inhibition mechanism was also done, suggesting the CI protonation and its further physical interaction with the Cl^–^ ion adsorbed on the metal surface. Finally, it was concluded that there was interaction between the lone electron pairs in the heteroatoms (N, S, and O) and π electrons from the aromatic ring with the Fe unoccupied d-orbital. A similar study was conducted by Shaban et al.^128^ who investigated the effect of the alkyl chain in three pyridinium cationic trimeric surfactants on the corrosion inhibition of API 5L X70 steel exposed to oil field formation water. The authors confirmed the dependence of the hydrophobicity on the alkyl chain increase by studying the critical micelle concentration (CMC) in both distilled and formation water. Additionally, a 3D image obtained by AFM was analyzed, and the presence of a more homogeneous API 5L X70 steel surface in the presence of **APMC18** than that of the sample without CI was confirmed. Afterward, the DFT analysis of the cations suggested that the HOMOs tended to be extended over the pyridinium rings toward the alkyl chain due to the increase in its length. A similar case occurred with **APMC12**. In contrast, with relatively shorter chains of (**APMC6**), the HOMO was only distributed over one alkyl chain. By MCs, the adsorption of the CIs on the metal surface related to the pyridinium rings and C=O was confirmed.

The inhibition mechanism proposed by these authors was similar to that in the previous case. However, the possible interactions between the cation and Br^–^ as well as the Fe backdonation to the π antibonding orbitals and the adhesion of the nonpolar hydrophobic alkyl chain that limited the interaction between the medium aggressive ions and steel surface were considered. As for Ortega-Vega et al.,^130^ these researchers studied the effect of the alkyl chain in the carboxylate anions of three PILs based on 2-hydroxiethylammonium on the corrosion inhibition process of API 5L X70 steel in 0.01 M NaCl. The same behavior pattern reported by Moselhy et al.^129^ was found who suggested that long alkyl chains increase the surface area occupied by the CI molecule on the steel surface. Another study on PILs employed as CIs was carried out by Likhanova et al.^131^ who investigated PILs with imidazolium imidazolate blocks as CIs for protecting API 5L X52 steel exposed to production water, calculating *IE*s of ∼80% at 100 ppm of PIL at 298 K. The authors observed that the PIL with 1-buty-3-vinylimidazolium displayed efficiencies of 56% from 10 ppm, which suggested that the combination of the PIL polymeric nature and the presence of analogue aromatic ring ions (imidazolium and imidazolate) contributed to the corrosion inhibition process of API 5L X52 steel. Likewise, three protection stages were proposed: (i) optimal accommodation of the PIL molecules, which implied the orientation of the CI toward the metal surface, achieving an average covered fraction of 75%; (ii) competition between molecules, where a decrease in the *IE* was provoked by the rearrangement of PIL molecules and their competition against molecules and ions in the water production corrosion environment; and finally, (iii) surface saturation, which implied that the API 5L X52 steel surface had limited adsorption and a slightly compact protecting film. In this very context, Ontiveros-Rosales et al.^132^ analyzed the corrosion inhibition process of API 5L X52 steel in production water by employing ILs based on a quaternary ammonium cation. It was found that at 100 ppm of trioctylmethylammonium dodecanedioate in production water, the corrosion of the metal surface was inhibited within an *IE* interval ranging from 67 to ∼87% in both laminar and transitory flow (N_RE_ = 500–2500). The displacements of the current density and corrosion potential denoted higher diffusion of the production water and IL ions in addition to the vortex momentum over the interphase of metal/inhibitor/corrosion products. The authors suggested that at this concentration, the ILs did not present steric hindrance to prevent their orientation toward the metal surface. Additionally, it was stated that the mass transfer of production water corrosive ions along with τ favored a synergistic effect on the IL inhibition process, even under hydrodynamic conditions. Finally, Chaudhry et al.^86^ analyzed an inorganic pigment based on nano- nickel ferrite (NiFe_2_O_4_) as potential CI for API 5L X80 steel in 3.5% NaCl.

Unlike the foregoing results, the *IE* dropped drastically with increasing C_INH_ in NaCl, which was associated with the possible electrocatalytic effect of the porous and permeable Ni and Fe film that worked as a galvanic cell, enabling Cl^–^ to dissolve the metal oxide film and its further attack of the Fe matrix, whereas Na^+^ facilitated the electron transfer. By this means, a wide variety of natural compounds and media (production water) or synthetic based on NaCl or brine (NACE) was found. Notwithstanding, in some cases, high concentrations (>100 ppm) were required to protect the API steel, mainly in the case of ILs, thus limiting the CIs that can be employed more efficiently in these media. In general, CIs with polycyclic aromatic compounds (pyrimidine, benzimidazole, pyridine, etc.) consist of at least one N or O heteroatom in their structure and/or functional groups (hydroxy, ketone, amine, ether, etc.) that participate as “anchoring” sites on the metal surface, which result in superior performance when competing against specific ions in each medium where they were evaluated (Cl^–^, CO_3_^2–^, HCO_3_^–^, HS^–^, O^2–^, F^–^, Br^–^, NO_3_^–^, PO_4_^3–^, SO_4_^2–^, etc.). Nonetheless, many of these CIs have been produced by organic synthesis without reporting a detailed analysis of their environmental impact despite their acceptable *IE* (>85%).

**Figure 3:**
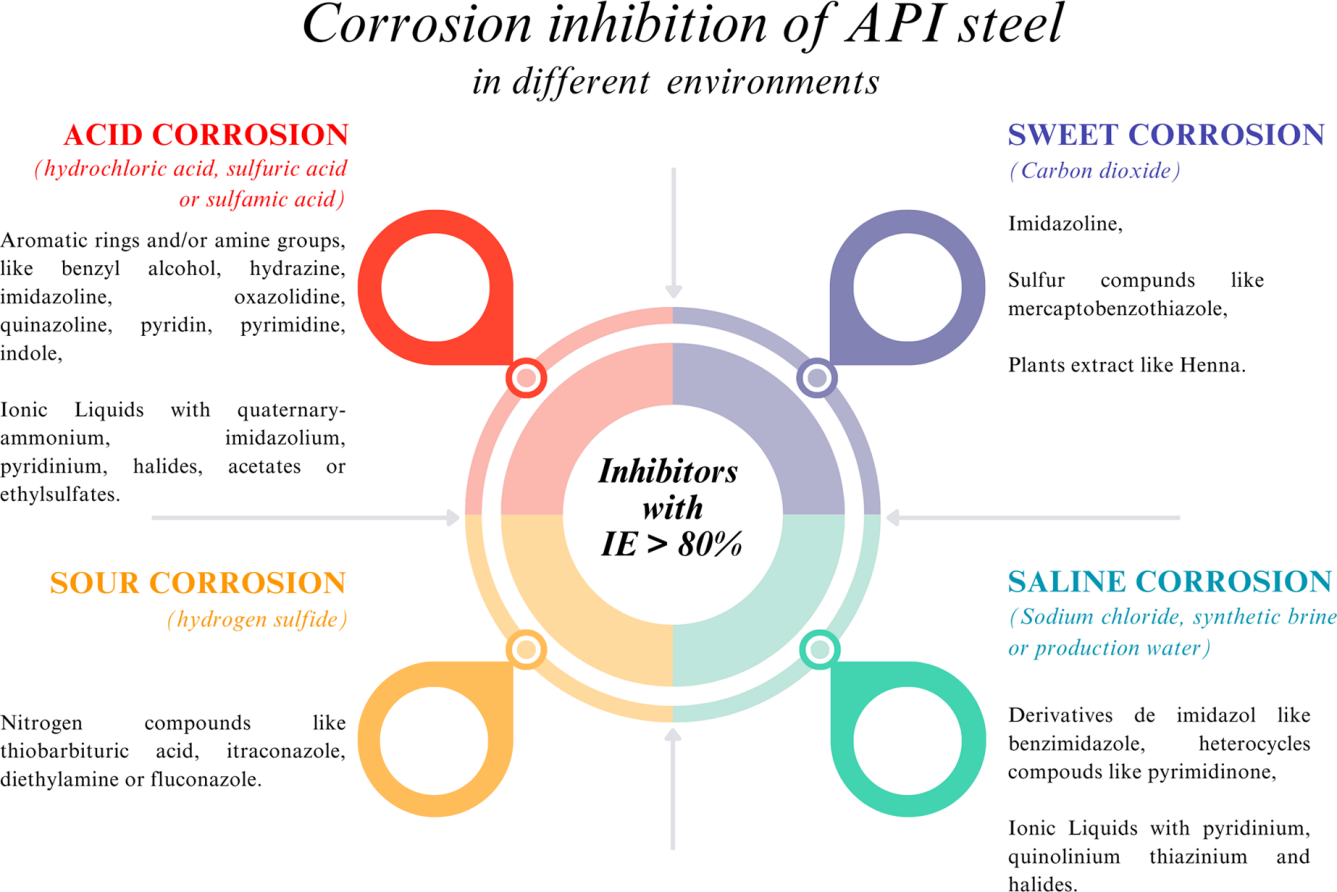
Examples of efficient CIs for the control of corrosion of API steels caused by various corrosive environments.

Throughout this review, it was found that the corrosion control of API steels promoted by acid, sweet, sour, and/or saline corrosive media is more interesting for the oil industry. For this reason, worldwide, numerous researchers have developed proposals with diverse chemical structures, which despite being different among them share the characteristic of having active centers that include different heteroatoms, mainly nitrogen and oxygen, more than one functional group such as amines, amides, carbonyls, etc. as well as aliphatic chains of different lengths that provide hydrophobic character. Figure 3 shows a brief list of CIs for API steel that have displayed a protecting effect above 80% when evaluated in different corrosive media. It is emphasized that CIs with these efficiencies contain molecules with rich electronic density and molecular size that form a protecting film and contribute to chemical adsorption processes. It should be noted that CIs with benzyl alcohol, hydrazine, oxazolidine, quaternary ammonium, or ethylsulfate-based ILs have been capable of inhibiting acid corrosion by HCl and H_2_SO_4_. In the case of sweet environment, nitrogen compounds like thiobarbituric acid, itraconazole, or diethylamine have been efficient CIs. In contrast, CIs with imidazoline, plant extracts, sulfur compounds like mercaptobenzothiazole, have displayed inhibiting behavior against sour corrosion. Finally, imidazole-derived CIs, pyrimidinone or quinolinium-based ILs, have been able to inhibit the corrosion of API steel in different saline media.

## 4 Conclusions

Through the present bibliographic revision, different CIs derived from inorganic and organic structures such as plant extracts, drugs, and synthetic compounds for API steels exposed to the main corrosive media of interest for the oil industry were analyzed.

In general, it was observed that C_INH_, temperature, immersion time, and flow rate are factors that affect the performance of CIs independently of the evaluated medium. Likewise, the presence of aromatic rings with π electrons, double bonds, and N, O, and S heteroatoms did not show a defined effect of the CIs that could allow the optimal design of CIs for API steels.

The variety of organic compounds produced from plant extracts is considered as a sustainable and environmentally friendly option; however, these compounds do not result feasible due to the fact that either high C_INH_ (>500 ppm) and/or the addition of synergistic agents are required in order to improve the inhibiting behavior and be considered as effective CIs (*IE*s above 90%). The same limitations apply for the chemical structures of different drugs and polymers obtained from natural sources. In contrast, some synthetic chemical compounds derived from triazole, benzyl, phenyl, pyrimidine, benzimidazole, benzothiazole, and imidazoline presented high *IE*s, notwithstanding their synthesis imply reagents and/or solvents that could inflict environmental damage in the long and short-term, which affects their application viability.

On the other hand, ILs derived mainly from ammonium and imidazole have been studied because of their great performance as CIs and in terms of the cation/anion selection and length of alkyl groups. In contrast, CIs with organic origin such as Ni_W_Zn_X_Fe_Y_O_Z_, CeO_2_, and Zn(NO_3_)_2_ displayed low *IE*s, which stemmed from the different species present in the composites or from their combination with organic species such as poly(acrylic acid).

Based on what has been reported in the literature, it can be suggested that CIs with just one heteroatom and asymmetric alkyl chains can be a feasible proposal in systems at low temperatures or under hydrodynamic conditions due to the structure simplicity. Mainly, it was observed that the presence of aromatic rings or carboxylic groups promotes steric hindrance that affects the molecular orientation during the adsorption process. In contrast, it can be inferred that the presence of various functional groups could allow the adsorption on multiple active centers on the metal surface, achieving longer CI adsorption times, even under temperature and flow effects. In the case of plant extracts, it is considered that the maximal profiting of the plant anatomy is an opportunity area because of the rich chemical composition and the identification of active compounds in the corrosion inhibition process of API steel is fundamental.

## Abbreviations

AFM: Atomic force microscopy
CI: Corrosion inhibitor
C_INH_: Inhibitor concentration
CR: Corrosion rate
DFT: Density functional theory
EFM: Electrochemical frequency modulation
EIS: Electrochemical impedance spectroscopy
FTIR: Fourier transform infrared
HOMO: Highest occupied molecular orbital
IE: Inhibition efficiency
*j*_corr_: Current density
LPR: Lineal polarization resistance
LUMO: Lowest unoccupied molecular orbital
MD: Molecular dynamics
MEP: Molecular electrostatic potential
MO: Molecular orbitals
N_RE_: Reynolds number
PDP: Potentiodynamic polarization
PILs: Poly ionic liquids
R_p_: Polarization resistance
SEM–EDS: Scanning electronic microscopy–energy dispersive spectroscopy
*T*: Temperature, [K]
*t*: Time, [h]
TGA: Thermogravimetric analysis
WL: Weight loss
XPS: X-ray photoelectron spectroscopy
τ: Shear stress
